# DNA methylation affects gene expression but not global chromatin structure in *Escherichia coli*

**DOI:** 10.1128/jb.00540-24

**Published:** 2025-07-14

**Authors:** Willow Jay Morgan, Haley M. Amemiya, Lydia Freddolino

**Affiliations:** 1 Department of Biological Chemistry, University of Michigan Medical School 12266 , Ann Arbor, Michigan, USA; 2 Cellular and Molecular Biology Program, University of Michigan Medical School 12266 , Ann Arbor, Michigan, USA; 3 Department of Computational Medicine and Bioinformatics, University of Michigan Medical School 12266 , Ann Arbor, Michigan, USA; University of Southern California, Los Angeles, California, USA

**Keywords:** DNA methyltransferase, bacterial chromatin, gene regulation, chromatin structure

## Abstract

The activity of DNA adenine methyltransferase (Dam) and DNA cytosine methyltransferase (Dcm) together account for nearly all methylated nucleotides in the *Escherichia coli* K-12 MG1655 genome. Previous studies have shown that perturbation of DNA methylation alters *E. coli* global gene expression, but it is unclear whether the methylation state of Dam or Dcm target sites regulates local transcription. In recent genome-wide experiments, we observed an underrepresentation of Dam sites in transcriptionally silent extended protein occupancy domains (EPODs), prompting us to hypothesize that EPOD formation is caused partially by low Dam site density. We thus hypothesized that a methylation-deficient version of MG1655 would show large-scale aberrations in chromatin structure. To test our hypothesis, we cloned methyltransferase deletion strains and performed global protein occupancy profiling using high-resolution *in vivo* protein occupancy display (IPOD-HR), chromatin immunoprecipitation for RNA polymerase (RNAP-ChIP), and transcriptome abundance profiling using RNA-seq. Our results indicate that loss of DNA methylation does not result in large-scale changes in genomic protein occupancy such as the formation of EPODs, indicating that the previously observed depletion of Dam sites in EPODs is correlative, rather than causal, in nature. However, loci with dense clustering of Dam methylation sites show methylation-dependent changes in local RNA polymerase and total protein occupancy, but local transcription is unaffected. Our transcriptome profiling data indicate that deletion of *dam* and/or *dcm* results in significant expression changes within some functional gene categories including SOS response, flagellar synthesis, and translation, but these expression changes appear to result from indirect regulatory consequences of methyltransferase deletion. In agreement with the downregulation of genes involved in flagellar synthesis, *dam* deletion is characterized by a swimming motility-deficient phenotype. We conclude that DNA methylation does not control the overall protein occupancy landscape of the *E. coli* genome and that observable changes in gene regulation generally do not result from the regulatory consequences of the local methylation states.

**IMPORTANCE:**

Previous studies of *E*. coli chromatin structure revealed a statistical association between the presence of silenced, highly protein-occupied regions of the genome and the depletion of modification sites for Dam methyltransferase. Here, we show that loss of DNA methylation does not substantively affect global chromatin structure in *E. coli*, thus demonstrating that the previously observed correlation was not causal. However, we observed specific methylation-dependent changes in gene expression, particularly affecting the SOS response, flagellar synthesis, and translation. These effects appear to be indirect regulatory consequences of methyltransferase deletion. Our work clarifies the role of methylation in chromatin structure and regulation, providing new insights into the mechanistic basis of gene expression and chromatin structure in *E. coli*.

## INTRODUCTION

DNA methylation in bacteria has well-supported roles in phage defense, chromosomal replication, DNA repair, and the regulation of gene expression (1). Most examples of bacterial methyltransferases are involved in restriction-modification phage defense systems, which involve the methylation of target sequences in bacterial DNA to protect against the endonuclease activity of restriction enzymes which are synthesized by the bacteria to cleave non-methylated phage DNA (2, 3). Methylated DNA generally results from the activity of methyltransferases, which cleave the methyl group from S-adenosyl-L-methionine (SAM) and transfer it to adenine or cytosine (4, 5). DNA methyltransferases have been found in a wide range of bacterial species to be involved in chromosomal maintenance, replication, DNA repair, cell cycle control, and virulence (6–13). There are also some examples of bacterial DNA methylation influencing local binding of regulatory proteins in promoters, which impact transcription (14–19), but it is unclear whether this is a common phenomenon in *E. coli* (1, 20–23). In the case of commonly studied *E. coli* K-12 laboratory strains, two major methyltransferases are known: Dam and Dcm.

DNA adenine methyltransferase (Dam) in *E. coli* catalyzes the formation of N^6^-methyladenine in the target motif 5′-GATC-3′ (24). Dam is conserved within Enterobacteriaceae. Over 99% of methylated adenines at ~19,000 sites in the genome of the *E. coli* K-12 strain MG1655 result from Dam activity (1). Dam is characterized as an orphan methyltransferase (25), as there is no known cognate restriction enzyme that cleaves at Dam methylation sites in *E. coli* K-12. Dam methylation has been implicated as a critical element of chromosomal maintenance, as the methylation state of ~11 Dam target sites at the origin of replication (oriC) regulates the initiation of chromosomal replication (26–28). During replication, Dam sites throughout the chromosome are processively methylated while lagging the replication fork, producing a transient hemimethylated state utilized by DNA repair machinery to differentiate between the template and newly synthesized strands in the event that mismatch repair is needed (29–32).

*E. coli* DNA cytosine methyltransferase (Dcm) methylates the inner cytosine in its 5′-CCWGG-3′ target motif (33), and Dcm is conserved within *Escherichia*. Dcm appears to be responsible for all cytosine methylation at ~12,000 sites in the *E. coli* K-12 genome (1, 22). Taken together with Dam, these two methyltransferases produce nearly the entire *E. coli* K-12 methylome (1, 34). Dcm is known to have a cognate restriction enzyme, *Eco*RII, which is not found in K-12 strains (35). The role of Dcm methylation in other cellular functions is less well-characterized than in the case of Dam. Although there are few to no changes in growth dynamics when *dcm* is deleted, there does appear to be a fitness benefit associated with Dcm methylation in the long-term stationary phase (22, 36). Dcm is also involved in Very Short Patch (VSP) repair in *E. coli* K-12, where the repair-associated endonuclease Vsr nicks double-stranded DNA at the Dcm target motif when the inner methylated cytosine is deaminated to thymine (5′-C**T**WGG-3′) (37). It is unclear whether the methylation state of the cytosine residue impacts this VSP repair process.

Perturbation of DNA methylation alters *E. coli* global gene expression to some extent, but the mechanisms by which the methylation state of Dam or Dcm sites regulates local transcription are not fully understood (20–22, 38, 39). In one example of DNA methylation acting as a transcriptional regulator, two nucleoid-associated proteins (NAPs) and Dam compete for binding to the promoter of the virulence-associated *pap* operon in uropathogenic *E. coli* (16, 17). NAPs are promiscuous DNA-binding proteins that confer chromosomal structure and act as global regulators (40, 41). There are other characterized examples of the methylation state at Dam motifs in NAP binding sites influencing NAP binding affinity—and in some cases, gene expression—across different bacterial strains (11, 18, 19, 42). A potential mechanism is that DNA methylation-dependent alterations of DNA-protein interactions result from the protrusion of the methyl group into the major groove, producing DNA curvature (39, 43, 44).

The full extent to which DNA methylation-altering NAP occupancy contributes to gene expression changes in *E. coli* K-12 is unknown. A recent study analyzing total protein occupancy data—produced by high-resolution *in vivo* protein occupancy display (IPOD-HR)—reported an underrepresentation of Dam target motifs in extended protein occupancy domains (EPODs) (45, 46). EPODs are ≥1 kilobase regions of the genome that have a continuously high protein occupancy signal; EPODs can be considered functional analogs to eukaryotic heterochromatin as EPODs are primarily formed by dense clusters of NAPs that coat DNA and silence local transcription (46, 47).

Our observation that Dam sites are underrepresented in EPODs—in addition to the regulatory cross-talk demonstrated with the *pap* operon and other examples—led us to speculate that there is a causal relationship between DNA methylation state and protein occupancy, which contributes to the formation of EPODs in *E. coli* K-12 MG1655. We thus hypothesized that a methylation-deficient version of MG1655 would show large-scale aberrations in chromatin structure (in particular, the formation and locations of EPODs), which might alter the regulation of silenced wild-type genomic regions. To test for such changes, we cloned single deletion mutants of Dam and Dcm (*Δdam* and *Δdcm*, respectively) and a double deletion mutant of both Dam and Dcm (*Δdam/Δdcm*), and we performed global protein occupancy profiling (using the IPOD-HR method (46) and transcriptome abundance profiling (using RNA-Seq) on these strains to produce global protein occupancy profiles and identify EPOD locations. Our results indicate that, relative to wild-type cells, DNA methylation-deficient mutants of *E. coli* K-12 MG1655 are not characterized by large-scale changes in genomic protein occupancy such as the formation of EPODs. Thus, the reduced abundance of Dam sites in EPODs does not cause EPOD formation (at least, not through reduced methylation density), but rather, is likely the consequence of some shared feature of these regions. However, we have identified a small number of loci with dense clustering of Dam methylation sites for which our data show methylation-dependent changes in local RNA polymerase and total protein occupancy. Our transcriptome profiling data indicate that deletion of *dam* and/or *dcm* results in significant expression changes within some functional gene categories including SOS response, flagellar synthesis, and translation, but these expression changes appear to result from indirect regulatory consequences of methyltransferase deletion rather than being due to perturbation of interactions between DNA methylation and regulatory proteins at gene promoters. As such, there are no changes in local transcription associated with the dense clusters of Dam sites. Dam deletion mutants were, however, characterized by a swimming motility-deficient phenotype, which is likely associated with the downregulation of genes involved in flagellar synthesis. Thus, we find that DNA methylation does not control the overall protein occupancy landscape of the *E. coli* genome and that changes in gene regulation are generally an indirect effect of loss of Dam methylation, rather than a direct regulatory consequence of the local methylation state.

## MATERIALS AND METHODS

### Bacterial strain construction

The “WT” parental strain of *E. coli* K-12 MG1655 was obtained from Dr. Haley Amemiya, who sourced it from Hani Goodzari (Tavazoie Lab, then at Princeton University) in 2009 as described in Amemiya et al. 2022 (47). This “WT” isolate is isogenic with ATCC 700926 except for an IS1 insertion in dgcJ (47, 48).

*Δdam, Δdcm*, and *Δdam/Δdcm* strains were constructed from the parental “WT” via P1 transduction of an FRT-flanked *kanR* marker from corresponding knockout strains in the Keio collection (49, 50). The *kanR* marker was excised through electroporating the pCP20 helper plasmid—which encodes for Flp recombinase—leaving a small scar in place of the indicated genes’ original open reading frames (51). Isolated transformants were grown overnight at 42°C to remove the temperature-sensitive pCP20, and these overnight cultures were non-selectively purified on LB plates grown overnight at 37°C. Candidate colonies were replica plated onto LB and selective plates to confirm the loss of the *kanR* marker and pCP20 plasmid. Sanger sequencing verified the deletion of the indicated gene with the replacement of a small scar.

*ΔlrhA* and *Δdam/ΔlrhA* strains were constructed through P1 transduction of FRT-flanked *kanR* from the corresponding Keio collection strain followed by pCP20-mediated recombination as described above.

All constructs were verified using Sanger sequencing through AZENTA Life Sciences GENEWIZ.

### Media and culture conditions

LB (Lennox) media (10 g/L tryptone, 5 g/L yeast extract, 5 g/L NaCl) was used for the above cloning, recovery of cryogenically preserved *E. coli* cells, and culturing for motility assays; 15 g/L bacteriological agar was added to the plates.

MOPS-RDM corresponding to the fully supplemented version of MOPS defined medium in Neidhardt et al. 1974 (52) (with 0.4% glucose as a carbon source) was used to grow *E. coli* cells for IPOD-HR, motility assays, and RNAP-ChIP; 15 g/L bacteriological agar was added to the MOPS-RDM recipe to make plates. Minimal MOPS media was made as specified in Neidhardt et al. 1974 (52) using 0.2% wt/vol glucose as a carbon source.

PYE (Peptone Yeast Extract; 2 g/L peptone, 1 g/L yeast extract, 1 mM MgSO_4_, pH 6.0) was used to grow *Caulobacter crescentus* cells to produce a spike-in reference for IPOD-HR. 20 g/L bacteriological agar was added for plates.

### Cell growth and harvest for IPOD-HR

Our procedures for IPOD-HR largely follow those described in Amemiya et al. 2022 (47). Cryogenically preserved cells were streaked onto a plate, and isolated colonies were subsequently grown in the same media as used for plating (MOPS-RDM for *E. coli*, PYE for *C. crescentus*) overnight at 37°C with shaking at 200 rpm. The culture was back-diluted into fresh, prewarmed media to an OD600 of 0.003 the next day. The culture was grown to the target OD600 of 0.2, and a 500 µL aliquot (for RNA-seq) was taken and added to 1 mL of RNAprotect Bacteria Reagent (Qiagen, Hilden, Germany) and preserved according to the manufacturer’s instructions. The remainder of the culture was treated with a final concentration of 150 µg/mL rifampicin and returned to the same growth conditions for another 10 min. The cultures were then rapidly pipetted into 50 mL conical tubes and mixed with concentrated formaldehyde/sodium phosphate (pH 7.4) buffer sufficient to yield a final concentration of 10 mM NaPO4 and 1% wt/vol formaldehyde.

Crosslinking proceeded for 5 min at room temperature with 300 rpm shaking and then quenched with an excess of glycine (final concentration 0.333 M) for 10 min with 300 rpm shaking at room temperature. Cells were then chilled on ice for 10 min and then pelleted and washed twice with ice-cold phosphate-buffered saline (PBS). The resulting pellets were dried by pipetting residual liquid, and the tubes were then snap-frozen in a dry ice-ethanol bath before being stored at −80°C.

### Cell lysis and DNA preparation

When resuspending frozen cell pellets, two pellets (taken from a single biological replicate) of each sample were separately resuspended with spike-in, sonicated, and then combined into one tube immediately after sonication. Individual frozen *C. crescentus* spike-in cell pellets were resuspended in 600 µL of 1× IPOD lysis buffer (10 mM Tris HCl, pH 8.0; 50 mM NaCl) containing 1× protease inhibitors (Roche Complete Mini, EDTA free, Roche Diagnostics GmbH, Mannheim, Germany) and 1.5 µL of ready-lyse (Epicentre, Madison, WI). The spike-in resuspension was used to resuspend one of the sample cell pellets, and then, the resuspended cells were incubated for 15 min in a 30°C water bath. Sonication was then performed on all samples using a Branson digital sonifier with a microtip at 25% amplitude for four pulses of 5 s with a 5-s rest between each pulse; samples were kept in an ice/water bath during sonication. The two separate tubes for each biological sample were then combined.

DNA digestion was performed by adding to the sonicated lysates 120 µg RNase A (Thermo Fisher Scientific, Waltham, MA), 12 µL DNase I (Fisher product #89835), 10.8 µL 100 mM MnCl2, and 9 µL 100 mM CaCl2, and then incubating on ice for 30 min. The digestion was quenched with 100 µL of 500 mM EDTA (pH 8.0), followed by clarification by centrifuge for 10 min at 13,000 rpm at 4°C. Aliquots were taken from the clarified lysate for IPOD-HR interface extraction, RNA polymerase chromatin immunoprecipitation, and cross-linking reversal and recovery of DNA as previously described (46). The procedures described in that reference were replicated here, apart from all the 2-min centrifuging steps instead of being done in 4 min.

For DNA recovery, standard phenol-chloroform extraction as ethanol precipitation as described in Ausubel F, 1998 (53), and the dried DNA was resuspended in 100 µL of TEe (10 mM Tris pH 8.0; 0.1 mM EDTA pH 8.0) for input samples, 20 µL of TEe for RNAP-ChIP samples, and 50 µL of TEe for IPOD samples.

### RNA isolation and sequencing preparation

RNA pellets were removed from −80°C and then immediately resuspended in 100 µL TE buffer (10 mM Tris pH 7.5; 1 mM EDTA). The resuspended pellet was treated with 1 µL lysozyme (Ready-Lyse; Lucigen, Ltd.) and incubated for 10 min at 4°C, followed by treatment with 10 µL proteinase K and incubation for 10 min at room temperature with vortexing every 2 min. RNA was then isolated using a Zymo RNA Clean and Concentrate 5 Kit twice for each sample, with a DNase digestion (25 µL eluate from first Zymo clean-up, 58 µL nuclease-free water, 10 µL 10X DNase Reaction Buffer, 2 µL RNase inhibitor, and 5 µL Baseline Zero DNase) at 37°C for 30 min in-between Zymo kit clean-ups. Samples were then ribo-depleted using a NEBNext rRNA Depletion (Bacteria) Kit according to the manufacturer’s instructions—with the exception that 10 µL of RNA sample, containing 1 µg of RNA, was used for probe hybridization. Following ribo-depletion, samples were again cleaned up with the Zymo Clean and Concentrate 5 Kit and then prepared for sequencing using the NEBNext Ultra II Directional RNA Sequencing Kit and then sequenced as described below for DNA samples.

### Preparation of next-generation sequencing (NGS) libraries

DNA samples were prepared for Illumina sequencing using NEBNext Ultra II Library Prep Kit (NEB product #E7103) and NEBNext Multiplex Oligos for Illumina (96 reactions, NEB product #E6442S). Deviations from the manufacturer’s directions to account for low average fragment sizes are described in Freddolino et al. 2021 (46). All libraries were sequenced on an Illumina NextSeq instrument.

Analysis of NGS data, read quality control and preprocessing, DNA sequencing and protein occupancy calling, and feature calling were performed as previously described in Freddolino et al. 2021 (46). However, we used here a more recent version of the IPOD-HR pipeline ipod_v2.5.7, which can be obtained from https://github.com/freddolino-lab/ipod (matching commit e2c2889 in that repository). Some of the software used in IPOD-HR version 2.5.7 includes cutadapt v3.5 (54), trimmomatic v0.39 (55), bowtie2 v.2.4.4 (56), and samtools v1.1.4 (57); definition files for building a singularity container exactly matching our workflow are available on the GitHub repository noted above.

A summary of the changes introduced between the IPOD-HR version utilized in Freddolino et al. 2021 (46) and the version utilized here are summarized as follows: following quantile normalization, each replicate of each data type (IPOD, ChIP, and input DNA) was median normalized to 100. A pseudocount of 0.25 was then added to each datum. Log_2_ ratios of IPOD or ChIP data relative to input data were calculated for each set of paired replicates. The log_2_ ratios were converted to robust z-scores and log_10_ *P*-values for visualization as described in Freddolino et al. 2021 (46); 95% confidence limits and mean estimates were calculated for log_2_ ratios, log_10_ *P*-values, and robust z-scores using jackknife sampling of the scores for all three biological replicates of each data type.

EPOD calling was performed similar to as described in Freddolino et al. 2021 (46) with the following deviations: EPOD seed regions were identified as any region at least 1,024 base pairs in length over which the median of a 768 bp rolling median exceeded the overall 90th or 75th percentile in the case of strict or loose EPOD calling, respectively, of a 256 bp rolling median over the entire chromosome. EPODs from all biological replicates of each given condition were “merged” into single, contiguous genomic intervals to assess the degree to which EPODs from replicate conditions overlap. EPODs were called separately at the biological replicate level, and then EPOD locations with low reproducibility were dropped from analysis based on an upper limit of 0.05 for the irreproducible discovery rate (58).

### Methylation motif flagging

To characterize the occupancy changes observed across genotypes relative to the methyltransferase target sites, we scanned each base pair of the *E. coli* U00096.3 genome and assigned each base pair a motif flag. Methyltransferase target sites were identified based on whether a sequence of base pairs matched the target motif of each methyltransferase: “GATC” for Dam and “CCTGG” or “CCAGG” for Dcm.

Scanning for these motifs was done using the motifs package from Biopython (59). The output of this Python v3.10.2 script was a listing of each methyltransferase target motif location in the genome. This information was used to create a file containing a list of every single base pair in the genome accompanied by an appropriate motif flag indicating membership to a methyltransferase target site.

### IPOD-HR and RNAP-ChIP occupancy at individual and clustered methylation sites

To capture more of the genomic context surrounding methylation sites, the slop command from bedtools v2.30 (60) was used to add 50 bp extensions to the start and end positions of each methylation site feature. The IPOD-HR and RNAP-ChIP occupancy scores at the extended methylation site features were found using bedtools intersect. Violin plots of the occupancy scores at methylation sites were made using seaborn v0.11.2 (61).

The density of methylation sites at genomic loci was determined by counting the number of methylation sites within each extended methylation site, and this count was then added as a flag to the extended methylation site.

Occupancy subtractions between genotypes were done to highlight occupancy changes unique in mutants relative to wild-type. To subtract occupancy between genotypes, negative values were adjusted to “0” for all genotypes, and then, wild-type occupancy was subtracted from each mutant occupancy track.

### Read end analysis

To identify the read ends of each input sample, “bedtools genomecov -ibam” with the “−5” and “−3” arguments was called on each BAM file output by the IPOD-HR alignment pipeline with the “-bg” argument used to produce bedgraph files. The 5’ and 3’ read ends were then combined into one file for each sample, and the read end count at each position was normalized by the total number of million read ends within each sample. A 100 base pair flank was added to each end of the “7 Dam Site” cluster 100 base pair windows, and bedtools intersect was used to find the normalized read end counts at these dense Dam site clusters. Heatmaps were generated using seaborn.

### EPOD analysis

Symmetrized overlap distance was calculated as shown in Equation 1 and as previously described in Amemiya et al. 2022 (47). Overlapping “strict” and “loose” EPODs were found using bedtools intersect. The frequency of Dam or Dcm sites in EPODs for each condition was calculated using bedtools intersect and then divided by the genomic total frequency of Dam or Dcm sites, respectively.


(1) $$1-\sqrt{A_{B}\times B_{A}}$$

**Equation 1:** Calculation of symmetrized overlap distance, which quantifies the overlap between two EPOD sets *A* and *B*, as previously described in Amemiya et al. 2022 (47). *X*_*Y*_ is the fraction of condition *Y*’s strict EPODs which are genomically overlapped (in at least one base pair position) with the loose EPODs in condition *X*.

For analysis of the representation of Dam sites in EPODs while controlling for the AT% of EPODs, each strict EPOD called for wild-type (“genomic EPODs”) was assigned to one of 10 evenly populated bins that were discretized based on AT content (AT%); 1,000 random shufflings of the EPOD genomic locations (“shuffled EPODs”), allowing the shuffled locations to overlap original EPOD locations (“overlaps”) or not (“no overlaps”), were produced using bedtools shuffle. The shuffled EPODs were then assigned to the AT% bins, and within each AT% bin, the Dam sites per kilobase for shuffled EPODs and genomic EPODs were compared by Poisson regression using R v4.1 (62) and plotted with ggplot2 v3.3.5 (63).

### Peak calling and analysis

To perform RNAP ChIP-seq peak calling, we first applied a 75 bp rolling mean to the RNAP ChIP-seq robust z-scores. Here, we term the resulting scores “smoothed scores.” Using a range of thresholds, we then considered a “peak” to be any contiguous region of the genome with a smoothed score above the given threshold. For every threshold, we calculated the Kullback-Leibler (KL) divergence between the distribution of smoothed scores in peak regions at the given threshold and the distribution of smoothed scores in an equal number of randomly selected non-peak regions of the genome of equal size to the peak regions. The threshold at which the maximum KL divergence between the peak smoothed score distribution and the non-peak smoothed score distribution was achieved was selected as the best threshold and was chosen for further analysis.

For comparing the occupancy peak profiles between strains, the locations of 2.0-threshold peak calls for each strain were compared using the symmetrized overlap distance calculation as described in Equation 1, but here, *X_Y_* is the fraction of condition *Y*’s 2.0-threshold peaks, which are genomically overlapped (in at least one basepair position) with any 2.0-threshold peaks in condition *X*. We note that the KL divergence landscapes for several of the data sets were virtually flat in the range of thresholds from 1.0 to 3.0 and chose 2.0 as a standard reference point suitable for all samples.

### RNA-seq analysis

The NGS data produced from Illumina sequencing of the RNA samples was processed through the IPOD-HR pipeline as described above up to but not including the alignment step. The Rockhopper v2.0.3 RNA-seq analysis system (64–66) was then utilized to align the processed RNA-seq reads to the U00096.3 genome and identify transcripts. Rockhopper returns q-values, which represent the statistical significance of the differential expression of each transcript between conditions. The log_2_-fold change in transcript values was calculated, with directionality assigned based on whether the mutant transcript value was greater (positive) or less (negative) than the wild-type transcript value; plots were generated using matplotlib v3.5.1 (67).

### iPAGE

We applied the iPAGE software previously described (68) to perform gene set enrichment analysis. To produce the data for running iPAGE, the q-values from the Rockhopper analysis described above, which compared the transcripts between our mutant and wild-type genotypes, were log_10_-transformed and assigned directionality (positive or negative) based on whether the transcript is higher (positive) or lower (negative) in abundance in the mutant genotype relative to wild-type. The directional log_10_q-values were fed into iPAGE as a continuous variable, and hence, iPAGE created equally populated discrete bins to rank the directional log_10_q-values and calculate their representation within each bin for all transcripts associated with a given GO term.

### Motility regulon expression analysis

The regulons of each regulator of *flhDC* were identified using the transcription factor to gene pairing database reported through RegulonDB (69). Expression changes between each of the methyltransferase mutants and wild-type were found using the log_2_ ratios of Rockhopper-derived transcript expression values for each regulator and each target. The degree to which changes in the expression of regulon components are consistent with the reported mode of action for each regulator-target pair (activator or silencer; dual regulators were ignored) was calculated by adjusting the sign of the log_2_-ratios (positive if the regulator’s mode of activity matches the expression change of the target, otherwise negative). The mean of directional log_2_-ratios was calculated to then evaluate the coherence of the regulator’s expression change with the concerted expression change across the regulator’s entire known regulon, here referred to as the “concerted log_2_-fold change in expression of regulon.” This value can then be compared with the log_2_-fold mutant versus wild-type expression change of the regulator for each regulon to assess whether there is evidence for a coherent increase or decrease in regulatory activity across a given regulon.

### Motility assays

Tryptone motility plates were made with 10 g/L of tryptone, 5 g/L of NaCl, and 3 g/L bacteriological agar, similar to those used in previously described motility assays (70). MOPS-RDM and MOPS-Minimal media were also prepared and turned into motility plates by adding 3 g/L bacteriological agar and otherwise following the recipes listed above. Plates were poured evenly by serological pipette (20 mL/plate) and left on the bench to dry overnight and then stored at 4°C the next day. Plates were not used if more than 2 months had passed since pouring them.

Cryogenically preserved cells were streaked out on standard LB-agar plates, and then, isolated colonies were grown in media of the same type for 12 h in the case of LB/Tryptone broth and MOPS-RDM and 24 h in the case of MOPS-Minimal. At the end of these growth periods, 1 mL of each culture was pelleted in a microcentrifuge for 3 min at 16,100 × *g* at 4°C. The supernatant was discarded, and the pellets were gently resuspended in 100 µL of sterile PBS pH 7.4. OD600 measurements were taken, and then, additional PBS volumes were added to each resuspended pellet to normalize all samples to 80% of the OD600 of the lowest sample. Any condensate on the lid of the plates was wiped off using a sterile replica plating velvet. A small filter disk soaked with 10 µL of 10 mM aspartic acid (as a chemoattractant) was then added to the center of MOPS-Minimal plates. One microliter of each OD-normalized sample was then spotted on all motility plates. We note to take care that the pipet tip should almost touch—but not break the surface tension—of the motility agar before dispensing the sample. After the plates were spotted, the plates were left facing up (media on bottom) and carefully parafilmed. The plates were then collectively placed into a Ziploc plastic bag containing some damp paper towels to prevent the drying of the plates. The bagged plates were then transferred to the 37°C incubator for at least 12 h before imaging at the first time point. After collecting the first time point, the plates were flipped upside-down (media on top) and were subsequently imaged every ~2 h.

To normalize the brightness of the motility plate images, the median brightness of each image was found using ImageMagick v7.1.04 (71) convert with the colorspace gray argument. The median of median brightness across all images was then calculated, and each image was adjusted in brightness to the value of the median of image-wise median brightness values using the ImageMagick convert evaluate Multiply argument.

### Growth curves

Using R v4.1 (62), we fit a linear model with log_2_OD600 as the dependent variable, time in hours as the independent variable, and an offset accounting for each replicate. Semilog plots were made using ggplot2 v3.3.5 (63).

## RESULTS

### Methyltransferase deletion globally perturbs expression of multiple large regulons

Although global protein occupancy is generally stable across most methylation sites when DNA methylation is perturbed, deletion of *dam* and/or *dcm* has been associated with some changes in gene expression (21, 22, 38, 72). We produced RNA-seq data, which were analyzed through the Rockhopper analysis suite (64–66) and found gene expression changes across many operons in the *Δdam* and *Δdam/Δdcm* genotypes, whereas the *Δdcm* strain showed relatively fewer significant changes in gene expression (Fig. 4). In the *Δdam* single mutant, the most positively expressed genes relative to wild-type are associated with DNA damage response, and the most downregulated genes are primarily members of the *gatYZABCDR* operon, which is involved in galactitol catabolism (73) (Table 1). Both the most upexpressed and most downexpressed genes in *Δdcm* encode gene products for transmembrane transport. Aside from the genes already represented in the single deletion mutants, the double deletion mutant *Δdam/Δdcm* is characterized by upregulation of maltooligosaccharide catabolism proteins encoded by *malP* and *malQ* (74), as well as downregulation of isoleucine and valine biosynthesis through *ivbL* (75).

**TABLE 1:** Transcripts showing the largest changes in abundance in each mutant genotype relative to wild-type, as calculated using Rockhopper

Five most significantly UP-expressed in *Δdam*								
Gene	Locus ID	Descriptive name	*Δdam*		*Δdcm*		*Δdam/Δdcm*	
			log2FC	−log10q	log2FC	−log10q	log2FC	−log10q
*ymfJ*	b1144	e14 prophage; uncharacterized protein YmfJ	+2.86	37.4	+0.58	0	+2.39	16.9
*recN*	b2616	DNA repair protein RecN	+2.56	26.1	+0.13	0.8	+2.66	27.57
*yebF*	b1847	secreted protein YebF	+2.34	122.11	+0.15	0.01	+2.3	109.68
*recX*	b2698	RecA inhibitor RecX	+2.21	21.81	+0.17	0	+2.36	28.93
*yebG*	b1848	DNA damage-inducible protein YebG	+2.19	92.77	+0.15	0.43	+2.26	96.61

5 most significantly DOWN-expressed in *Δdam*								
Gene	Locus ID	Descriptive Name	*Δdam*		*Δdcm*		*Δdam/Δdcm*	
			log2FC	−log10q	log2FC	−log10q	log2FC	−log10q
*gatA*	b2094	galactitol-specific PTS enzyme IIA component GatA	−3.13	143.99	−1.35	18.8	−2.6	62.54
*yahA*	b0315	DNA-binding transcriptional dual regulator/c-di-GMP phosphodiesterase PdeL	−3.08	219.99	+0.45	0	−2.79	90.75
*gatB*	b2093	galactitol-specific PTS enzyme IIB component GatB	−2.89	48.02	−1.21	7.12	−2.89	33.77
*gatZ*	b2095	putative tagatose-1,6-bisphosphate aldolase two chaperone GatZ	−2.5	132.4	−0.97	9.77	−2.37	213.13
*gatY*	b2096	tagatose-1,6-bisphosphate aldolase 2 Gat Y	−2.38	110.32	−1	15.01	−2.24	208.56

Five most significantly UP-expressed in *Δdcm*								
Gene	Locus ID	Descriptive Name	*Δdam*		*Δdcm*		*Δdam/Δdcm*	
			log2FC	−log10q	log2FC	−log10q	log2FC	−log10q
*rsmI*	b3146	16S rRNA 2′-O-ribose C1402 methyltransferase	−0.23	1.93	+1.55	1.43	1.4	0.85
*yjcB*	b4060	uncharacterized protein YjcB	+0.62	0.25	+1.13	1.44	0.69	0.59
*sugE (gdx*)	b4148	guanidinium exporter	+0.52	1.13	+0.98	3.8	1.06	5.97
*yrbL*	b3207	protein kinase-like domain-containing protein YrbL	+0.48	1.64	+0.83	4.67	0.48	2.53
*azuC*	b4663	small regulatory membrane protein AzuC	+0.43	1.27	+0.81	4.4	0.15	0.03

Five most significantly DOWN-expressed in *Δdcm*								
Gene	Locus ID	Descriptive Name	*Δdam*		*Δdcm*		*Δdam/Δdcm*	
			log2FC	−log10q	log2FC	−log10q	log2FC	−log10q
*yjiY*	b4354	pyruvate:H + symporter	−1.27	16.55	−1.92	38.8	−2.03	72.61
*ilvC*	b2774	ketol-acid reductoisomerase (NADP+)	−1.59	27.03	−1.63	29.62	−1.33	41.85
*mglB*	b2150	D-galactose/methyl-galactoside ABC transporter periplasmic binding protein	−0.78	1.78	−1.58	15.9	−0.68	0.48
*gatA*	b2094	galactitol-specific PTS enzyme IIA component	−3.13	143.99	−1.35	18.8	−2.6	62.54
*glpT*	b2240	sn-glycerol 3-phosphate:phosphate antiporter	−0.04	0	−1.31	15.74	−0.12	3.45

five most significantly UP-expressed in *Δdam/Δdcm*								
Gene	Locus ID	Descriptive Name	*Δdam*		*Δdcm*		*Δdam/Δdcm*	
			log2FC	−log10q	log2FC	−log10q	log2FC	−log10q
*malP*	b3417	maltodextrin phosphorylase	+0.84	4.59E-03	−0.16	1.44	+2.84	35.85
*recN*	b2616	DNA repair protein RecN	+2.56	26.1	+0.13	0.8	+2.66	27.57
*malQ*	b3416	4-α-glucanotransferase	+0.67	0	−0.21	1.34	+2.62	25.83
*ymfJ*	b1144	e14 prophage; uncharacterized protein YmfJ	+2.86	37.41	+0.58	0	+2.39	16.91
*recX*	b2698	RecA inhibitor RecX	+2.21	21.81	+0.17	0	+2.36	28.93

Five most significantly DOWN-expressed in *Δdam/Δdcm*								
Gene	Locus ID	Descriptive Name	*Δdam*		*Δdcm*		*Δdam/Δdcm*	
			log2FC	−log10q	log2FC	−log10q	log2FC	−log10q
*gatB*	b2093	galactitol-specific PTS enzyme IIB component	−2.89	48.025	−1.21	7.12	−2.89	33.77
*yahA*	b0315	DNA-binding transcriptional dual regulator/c-di-GMP phosphodiesterase PdeL	−3.08	219.99	+0.45	0	−2.79	90.75
*gatA*	b2094	galactitol-specific PTS enzyme IIA component	−3.13	143.99	−1.35	18.8	−2.6	62.54
*gatZ*	b2095	putative tagatose-1,6-bisphosphate aldolase 2 chaperone	−2.5	132.4	−0.97	9.77	−2.37	213.13
*ivbL*	b3672	ilvBN operon leader peptide	−2.03	34.378	−0.81	4.74	−2.25	46.08

**Fig 1:**
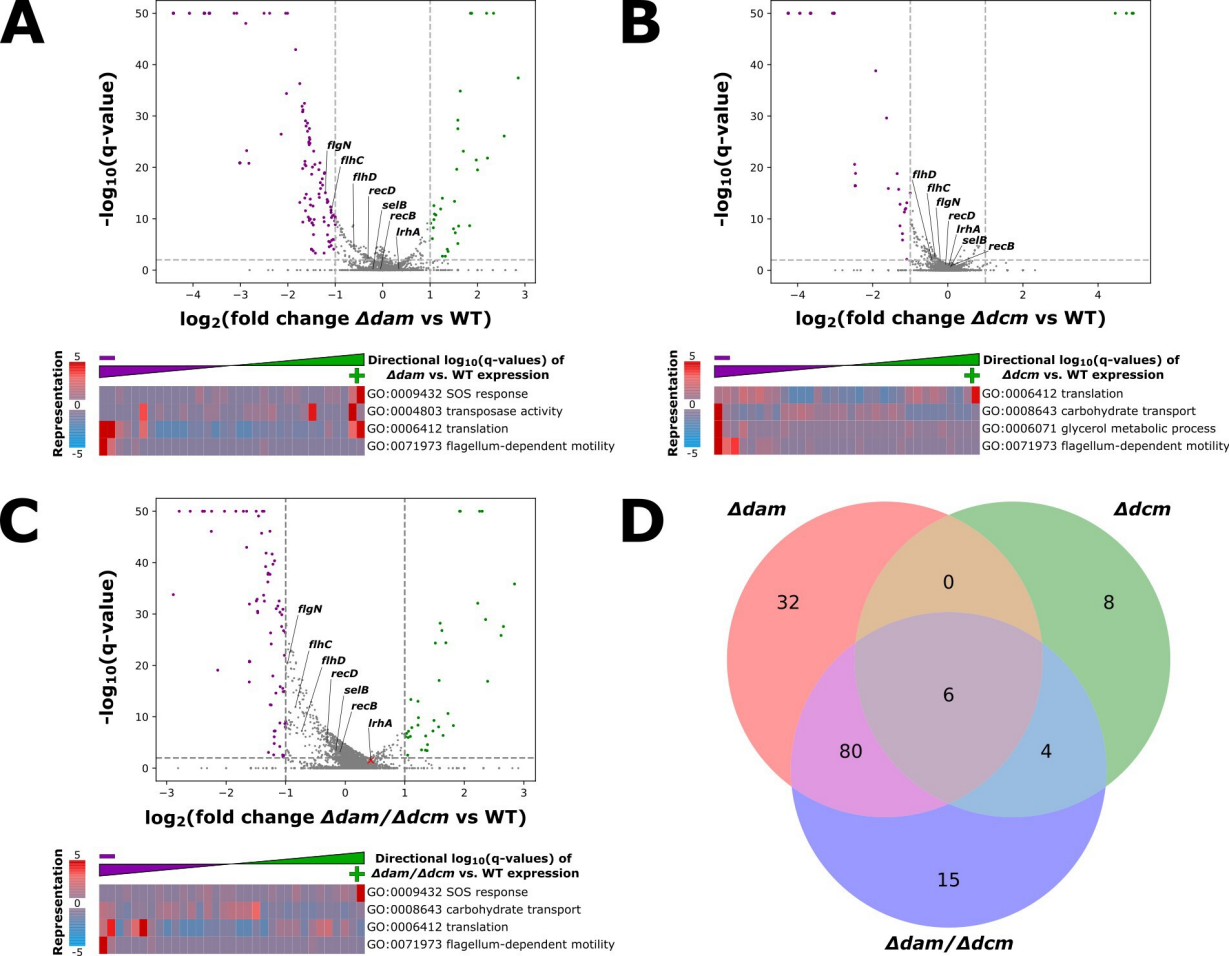
(**A**) *Δdam*, (**B**) *Δdcm,* and (**C**) *Δdam/Δdcm* versus wild-type change in expression of genes. Purple genes are reduced in transcript abundance, whereas green genes are increased in abundance, in the mutant relative to wild-type. Some genes of interest are labeled by name. Below each volcano plot is shown gene set enrichment analysis for RNA-seq data across the indicated genotype (relative to wild type) for a selected subset of gene ontology terms. iPAGE reports the representation of directional log10(q-values) across the genes annotated with each Gene Ontology (GO) term—thus, a redder bin indicates an over-representation of genes from the specified GO term (row) at that expression change bracket (column). (**D**) Venn diagram of significantly differentially expressed (log-fold change ≥1.0 or ≤−1.0; and FDR-corrected *P*-value ≤ 0.01) genes for each mutant genotype relative to wild-type.

We then performed gene set enrichment analysis on our RNA-seq data, which further supports gene expression changes across multiple gene ontology (GO) categories in methyltransferase deletion mutants (Fig. 1). We found a decrease in the expression of several genes associated with flagellum-dependent motility, which was consistent in all methyltransferase mutants relative to wild-type. Previous findings that activation of the SOS response, which is associated with DNA damage repair, occurs upon *dam* deletion were also reproduced here, likely caused by interference with hemimethylation-dependent mismatch repair and/or perturbation of normal replication initiation (21, 76). We also detected differential expression of gene products involved in maintaining transposons across all methyltransferase mutants, which supports previous findings that *dam* methylation impacts transposase expression and transposition activity (77, 78). Genes relating to translation and amino acid biosynthesis have also been reported to significantly change in expression in *Δdam* strains (20, 79), and our methyltransferase mutants all show substantial expression changes in translation and amino acid biosynthesis (Fig. S1). However, the mechanistic basis underlying the relationship between gene expression and DNA methylation in *E. coli* K-12, including the extent to which DNA methylation impacts the binding of transcription regulatory proteins, has yet to be elucidated.

### Dam sites are statistically depleted in extended protein occupancy domains while controlling for AT content

We previously found, in wild-type *E. coli* K-12 MG1655, that there is a statistically significant underrepresentation of Dam sites within regions of the genome covered by EPODs relative to non-EPOD regions (46). To control for the difference in AT% between EPODs and non-EPOD regions and address the possibility that the depletion of Dam sites in EPODs is caused by the AT-richness of EPODs, we compared the frequency of Dam sites within EPODs to the frequency of Dam sites at other loci of the same length as each EPOD (“shuffled” EPODs). We assigned each wild-type EPOD (“genomic” EPODs) to 10 equally populated bins defined by AT%, assigned 1,000 permutations of shuffled EPODs to these AT% bins, and performed Poisson regression analysis with terms for “genomic” (real) versus “shuffled” EPODs as well as AT% bin membership. Our results show that “genomic” EPODs contain significantly fewer Dam sites than “shuffled” EPODs while incorporating for the AT% bin membership term, and this is true both when shuffled EPODs are restricted from being shuffled to the positions originally occupied by genomic EPODs (−0.069 regression coefficient estimate for “genomic” EPODs, −0.120 to −0.017 95% CI, 0.0104 *P*-value) and when shuffled EPODs are permitted to overlap genomic EPOD positions (−0.110 coefficient estimate for “genomic” EPODs, −0.170 to −0.064 95% CI, 1.6 × 10^−5^ *P*-value). We performed this same Poisson regression analysis for Dcm sites and found no sign of anticorrelation for when we do not permit “shuffled” EPODs to overlap “genomic” EPODs (−0.011 coefficient estimate for “genomic” EPODs, −0.072 to 0.050 95% confidence interval, 0.734 *P*-value) and statistically significant anticorrelation—but much weaker than the anticorrelation for Dam sites—when we do allow “shuffled” EPODs to overlap “genomic.” EPODs (−0.065 coefficient estimate, −0.130 to −0.004 95% CI, 0.038 *P*-value). These results show that even after controlling for AT%, the “genomic” EPODs have significantly fewer Dam sites than expected by random chance, thus reinforcing the basis of our hypothesis that there may be an association between EPOD formation—and more generally, protein occupancy—and DNA methylation (i.e., that the presence of Dam methylation might inhibit the NAP binding that gives rise to EPODs).

### Loss of DNA methylation minimally alters protein occupancy on the *E. coli* K-12 MG1655 genome

To characterize a set of protein binding events that may be dependent on DNA methylation state, we utilized the IPOD-HR methodology to profile changes in the global protein occupancy of the *E. coli* K-12 MG1655 chromosome when either or both of the genes encoding the two primary methyltransferases, *dam* and *dcm*, are deleted. IPOD-HR has been previously described and applied to characterize global protein occupancy changes in MG1655 NAP deletion mutants (46, 47). The IPOD-HR methodology involves crosslinking and Illumina sample preparation like other protein-DNA extraction and enrichment methodologies such as chromatin immunoprecipitation followed by sequencing (ChIP-seq) (80). To produce a global profile of all protein occupancy across the genome, IPOD-HR utilizes physicochemical principles to enrich crosslinked protein-DNA complexes at an aqueous-organic interface during phenol/chloroform extraction. ChIP for RNA polymerase (RNAP-ChIP) is also performed on the same biological samples as used for IPOD-HR to remove the RNAP signal from the IPOD-HR occupancy profile. Producing RNAP-ChIP data allows us to isolate changes in occupancy of RNA polymerase (which we have observed to be correlated with gene expression changes), and the subtraction of the RNAP-ChIP signal from the IPOD-HR total protein signal highlights changes in transcription factor and NAP occupancy that might otherwise be obscured by replacement with RNAP (46). Our use of rifampicin prior to crosslinking results in RNAP-ChIP peaks that correspond to sites of holoenzyme recruitment, and previous analysis of RNAP-ChIP data produced by our methodology has shown a strong correlation between transcript levels and RNAP-ChIP signal in corresponding promoter regions (46). We also performed RNA-seq in parallel with IPOD-HR and RNAP-ChIP on *E. coli* MG1655 (WT) and our methyltransferase deletion mutants (*Δdam, Δdcm*, and *Δdam/Δdcm*) to characterize changes in gene expression that may result from changes in protein occupancy when DNA methylation is perturbed.

**Fig 2:**
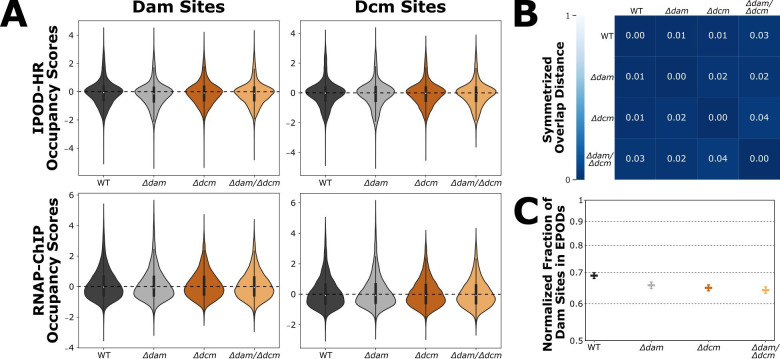
**(A**) Distribution of IPOD-HR and RNAP-ChIP occupancy scores in genomic windows centered on each Dam (104 bp window) or Dcm (105 bp window) target motif. (**B**) Symmetrized Overlap Distances were calculated as described in Amemiya *et al.*, 2022 (47) to assess the similarity in global EPOD composition between strains. A value of 0 indicates that all EPODs between the two strains overlap. (**C**) Normalized fraction of total Dam sites found within EPODs for each strain. The fraction of Dam sites within EPODs was normalized by the fraction of total genomic base pairs covered by EPODs, and 95% confidence intervals were calculated through jackknife resampling of the EPOD genomic positions. The y-axis is log-scaled.

To explore the possibility of a global change in protein occupancy local to DNA methylation sites when the primary DNA methyltransferases are deleted, we first identified every potential Dam or Dcm site based on the appearance of their target motifs (5′-GATC-3′ or 5′-CCWGG-3′, respectively) in the *E. coli* K-12 MG1655 U00096.3 sequence. To capture the genomic context around each methylation site, we captured the 50 base pairs (bp) both upstream and downstream of each Dam or Dcm site, which generated 104 bp windows centered on each Dam site and 105 bp windows centered on each Dcm site. An association between methylation state and protein occupancy was then made by creating a distribution of the means of IPOD-HR or RNAP-ChIP occupancy scores within each methylation site window and comparing across genotypes (Fig. 2A). We applied the one-sample version of the Bayesian Estimation Supersedes the *t*-test (BEST) analysis method to generate 95% credible intervals, which for all conditions had a range of less than 0.1, and found that the distribution of occupancy scores at methylation sites is stable across genotypes—although there are global changes in features of RNAP-ChIP occupancy (Fig. S3). Despite some global changes in protein occupancy resulting from methyltransferase deletion, we generally do not observe substantial differences in local IPOD-HR or RNAP-ChIP protein occupancy across all DNA methylation sites when *dam* and/or *dcm* are deleted.

The statistical underrepresentation of Dam sites in EPODs also motivated us to explore how EPOD locations change relative to DNA methylation sites when *dam* and/or *dcm* are deleted. To characterize the set of EPOD locations for each genotype, EPOD calling was performed on IPOD-HR data as previously reported with some minor modifications (as noted in Methods) (46). Symmetrized overlap distances (SODs) were calculated as previously described in Amemiya et al. 2022 (47) to analyze similarity in the set of EPOD locations between genotypes (Fig. 2B). A SOD score of “0” represents perfect overlap of EPOD locations between sets, and a score of “1” represents zero overlap in EPOD locations between sets. Given that the SOD scores between any compared genotypes were 0.04 or lower, we observe negligible changes in the set of EPOD locations when *dam* and/or *dcm* are deleted. We additionally found no substantial change between genotypes in the fraction of Dam or Dcm target sites within EPODs (Fig. 2C), although a slight decrease in the fraction of Dam sites in EPODs is apparent. These results indicate that there are few to no changes in large-scale protein occupancy features, such as EPOD formation, when DNA methylation is perturbed through methyltransferase deletion.

To evaluate whether the loss of methylation signals might still alter gene expression within EPODs when compared with non-EPOD regions of the genome, we performed RNA-seq experiments on all four strains included in our study and calculated the median mutant versus wild-type log_2_FC of transcripts within EPODs and subtracted the median mutant versus wild-type log_2_FC of transcripts outside of EPODs. We found a small but significant decrease in median expression of transcripts within EPODs versus outside of EPODs when *dam* and both *dam* and *dcm* are deleted, but there is no difference in median expression between within and outside of EPODs when just *dcm* is deleted (median difference of mutant versus wild-type log_2_FC of transcripts within EPODs minus outside of EPODs and Wilcoxon two-sided rank-sum p-value: 0.06 and 1.1 × 10^−5^ for *Δdam*, 0.00 and 0.95 for *Δdcm*, 0.15 and 3.2 × 10^−8^ for *Δdam/Δdcm*). Thus, there is a small relative decrease in transcription inside vs. outside of EPODs when the *dam* is deleted, although especially considering the absence of systematic occupancy changes, the mechanism and biological significance of these changes remain unclear.

### Protein occupancy signal in *dam* deletion mutants is decreased at dense clusters of Dam target sites

**Fig 3:**
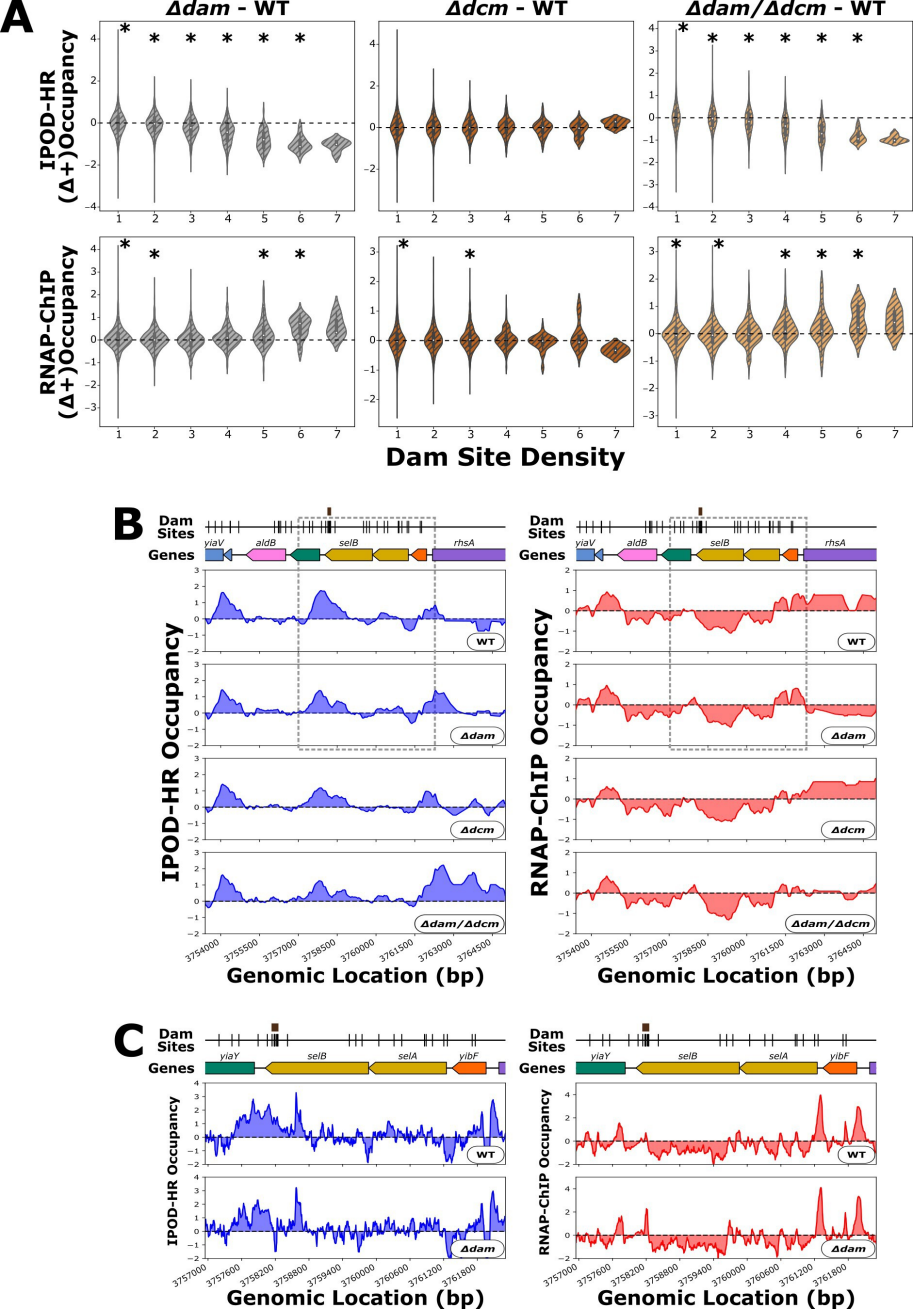
(**A**) Distribution of changes in mean IPOD-HR or RNAP-ChIP occupancy scores in 104 bp windows centered on each Dam target motif; positive scores indicate higher occupancy in the indicated mutant relative to WT. "Site Density" on the x-axis refers to the number of Dam sites within each window. Asterisks represent *P*-values of <0.01 by Wilcoxon signed rank test with Bonferroni correction. At the higher site densities (6 and 7 site densities), there is a lack of statistical power due to a small number of loci with such high methylation site densities. (**B**) Genomic context of *selB* showing 512 bp rolling mean of IPOD-HR (blue occupancy trace) or RNAP-ChIP (red occupancy trace) robust z-scores. Brown boxes above markers on the “Dam Sites” tracks indicate “7 Dam Site Density” clusters of interest. Genes are differentially colored based on their membership to functional gene clusters. The dashed box designates the locus which is shown in panel C. (**C**) Zoomed in view of *selB* (corresponding to the boxed region of panel B) showing IPOD-HR (blue occupancy trace) or RNAP-ChIP (red occupancy trace) robust z-scores.

Considering that multiple DNA methylation events in close genomic proximity could induce a greater degree of DNA curvature (44), we hypothesized that genomic regions with dense clusters of methylation sites may experience more pronounced protein occupancy changes when DNA methylation is perturbed. In addition, it has been shown that certain DNA-binding proteins such as SeqA preferentially bind to regions with multiple proximal Dam sites (21, 81). These considerations led us to consider how the change in IPOD-HR occupancy differences between methyltransferase deletion mutants and the wild type might vary as a function of local methylation site density. Here, we observe a negative correlation between mutant-specific IPOD-HR signal and Dam Site Density when *dam* is deleted (Fig. 3A). In other words, we find less total protein occupancy at dense clusters of Dam sites when *dam* is deleted, whereas locations with lower Dam site densities are unaffected, as are Dcm sites (Fig. S3).

Our analysis defined twelve unique loci containing a Dam site annotated with a Dam Site Density of 6 as well as three unique loci with a Dam site annotated with a Dam Site Density of 7; we refer to these regions collectively as “high-density Dam site clusters.” Each of these high-density Dam site clusters appears within ORFs and is thus absent from promoters or intergenic regions. Despite the *Δdam*-associated increase in RNA polymerase at most of these high-density Dam site clusters, there is only one locus that presented with a significant change in proximal gene expression, which is the *oriC-*adjacent gene *mnmG* (Rockhopper q-values for transcript count of *mnmG*: ~0.0017 for *Δdam* vs. WT, ~1.0 for *Δdcm* vs. WT, ~0.00036 for *Δdam/Δdcm* vs. WT). Overall, it appears that the *Δdam*-dependent change in RNAP and total protein occupancy at high-density Dam site clusters are not impacting the expression of known local transcripts (see GEO data set GSE279866).

One locus of interest featuring a cluster of seven Dam sites is at the terminal end of the *selB* coding region (Fig. 3B and C). Here, we observe *Δdam*-dependent peaks in RNAP occupancy both at the Dam site cluster as well as at the promoter immediately upstream of *selB*. However, transcript levels of *selB* do not change substantially in *Δdam* genotypes (Rockhopper mutants versus wild-type log_2_FC / q-values of *selB*: −0.21/0.69 for *Δdam* vs. WT, 0.072/0.27 for *Δdcm* vs. WT, −0.16/3.6 × 10^−4^ for *Δdam/Δdcm* vs. WT). The gene with a promoter immediately downstream of the *Δdam*-dependent RNAP-ChIP peak at the *selB* Dam site cluster, *yiaY*, is not transcribed in any of our genotypes (see GEO data set GSE279866). There is a decrease in IPOD-HR signal associated with the increase in RNAP-ChIP signal at the *selB* Dam site cluster in *Δdam* strains, but we cannot find any reports on what protein may bind to this region.

**Fig 4:**
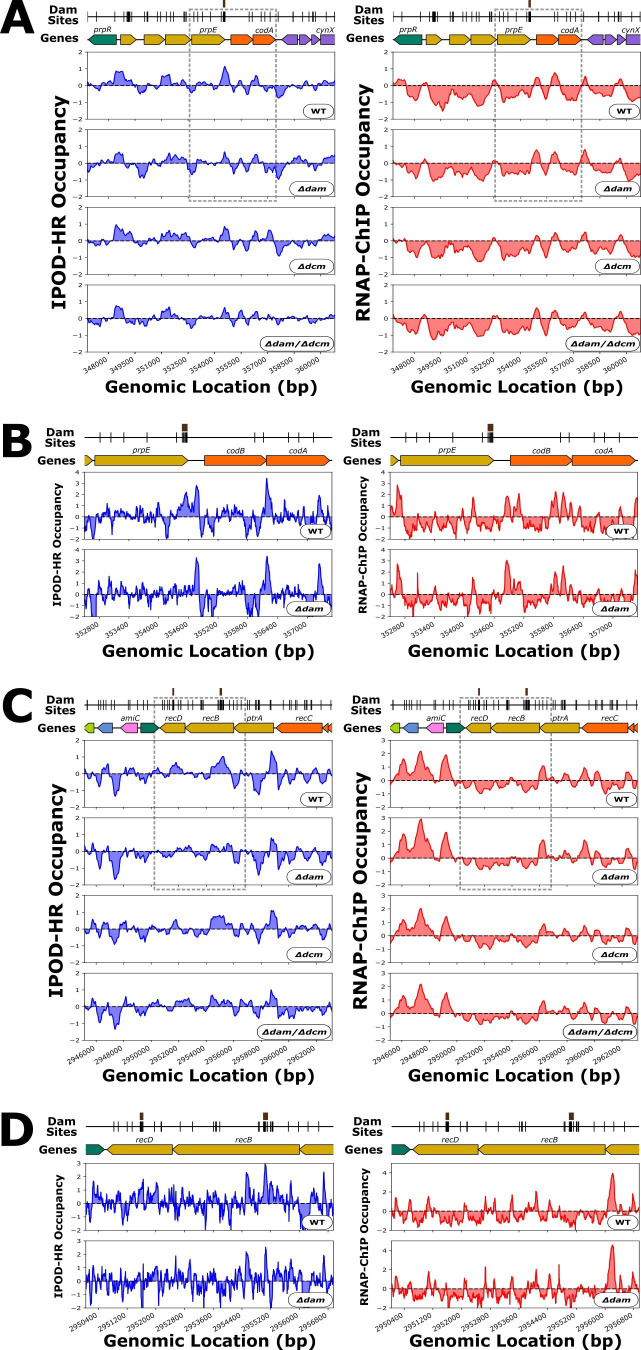
(**A**) Genomic context of *prpE* showing 512 bp rolling mean of IPOD-HR (blue occupancy trace) or RNAP-ChIP (red occupancy trace) robust z-scores. Brown boxes above markers on the “Dam Sites” tracks indicate “7 Dam Site Density” clusters of interest. Genes are differentially colored based on their membership to functional gene clusters. The dashed box designates the locus which is shown in panel B. (**B**) IPOD-HR (blue occupancy trace) or RNAP-ChIP (red occupancy trace) robust z-scores in the immediate vicinity of *prpE*. (**C**) As in panel A, showing the genomic context around *recBD*. (**D**) As in panel B, for the boxed region indicated in panel D.

We also identified a 7 Dam site cluster at the terminal end of *prpE* (Fig. 4A and B), which is comparable with the *selB* case in that it features an increase in RNAP-ChIP signal and a decrease in IPOD-HR signal in our *Δdam* genotypes. In contrast to the *selB* case, for *prpE,* the *Δdam*-dependent peak in RNAP-ChIP appears ~200 basepairs downstream of the Dam site cluster, and hence, the RNAP-ChIP peak is proximal to the promoter for *codB*, which shows modest but not statistically significant increases in transcript levels in all of our methyltransferase deletion strains (Rockhopper mutants versus wild-type log_2_FC / q-values of *codB*: 0.30/1.0 for *Δdam* vs. WT, 0.45/1.0 for *Δdcm* vs. WT, 0.31/0.11 for *Δdam/Δdcm* vs. WT). Given that there is a similar magnitude of upregulation of *codB* in *Δdcm* compared with *Δdam*, the upregulation of *codB* may not actually be associated with the *Δdam*-dependent increase in RNAP-ChIP, but rather, it is likely statistical noise. *prpE* is not actively transcribed in any of our strains (see Supplementary figures).

There are two proximal 7 Dam site clusters within the *recB* and *recD* coding regions (Fig. 4C and D). We find this notable because of the involvement of *dam*, *recB*, and *recD* in *E. coli* DNA mismatch repair, and the fact that previous attempts to delete both *dam* and *recB* found such a strain to be inviable (82). Despite an increase in the RNAP-ChIP signal at the *recBD* promoter in our *Δdam* genotype, the transcript levels of *recB* in fact show a small decrease in our *dam* mutant strains (Rockhopper mutants versus wild-type log_2_FC / q-values of *recB*: −0.043/0.65 for *Δdam* vs. WT, 0.082/0.14 for *Δdcm* vs. WT, −0.087/7.7 x 10^−4^ for *Δdam/Δdcm* vs. WT) and *recD* (Rockhopper mutants versus wild-type log_2_FC / q-values of *recD*: −0.31/0.0052 for *Δdam* vs. WT, −0.057/0.075 for *Δdcm* vs. WT, −0.31/7.1 × 10^−5^ for *Δdam/Δdcm* vs. WT). The *recD* Dam site cluster is in the middle of the gene body, whereas the 7 Dam site cluster in *recB* is a few hundred base pairs downstream of a putative *recB* promoter (defined by the presence of an RNA polymerase ChIP peak), and the IPOD-HR occupancy at both sites is substantially decreased in our *Δdam* genotype.

Rifampicin—at the concentration added to our cells used for RNAP-ChIP—prevents promoter clearance, which leads to a build-up of RNAP at active promoters (83, 84). We thus hypothesized that the *Δdam*-dependent RNAP-ChIP peak observed at these Dam site clusters may result from RNAP that has been directly recruited for transcription—perhaps of a small RNA. However, there does not appear to be any increase in RNA-seq reads local to the RNAP-ChIP peaks at these Dam site clusters in *Δdam* strains (Fig. S4). We also considered that these RNAP-ChIP peaks may result from RNAP being stalled, possibly at a Dam methylation-directed repair site due to the increase in DNA damage and upregulation of SOS response in *Δdam* strains (30, 79, 85). To investigate the possibility of DNA damage, we produced heatmaps of the normalized frequency of read ends at some of the seven density Dam site clusters, but none of the heatmaps show a *Δdam*-dependent pattern in read end accumulation local to the RNAP-ChIP peaks (Fig. S5), indicating no clear signature of increased strand breaks near the Dam site cluster (although any such accumulation may well have been obscured anyway by the fragmentation steps inherent to our purification protocols). Taken together, these results indicate that RNAP may be directly recruited to—but not actively transcribing—the high-density Dam site clusters in *Δdam* strains, or RNAP may be stalled but not because of DNA strand breaks; additional investigation would be required to distinguish between these possibilities.

### Previously identified instances of local methylation-sensitive regulation are recapitulated in our data

To systematically investigate the concordance of our data with previous reports of correlations between methylation and protein occupancy in *E. coli* strains (18, 19, 22, 86–89), we first reviewed the available literature to compile a list of candidate genes, which had previously been studied in the context of potential contributions of Dam methylation to their cis-regulatory logic, requiring that those genes had (i) been investigated for change in transcript level based on methylation state of immediately upstream Dam or Dcm sites or (ii) had been reported as having persistently unmethylated immediately upstream Dam or Dcm motifs (Table 2). We included data arising both from genetic deletions of *dam* or *dcm*, and from 5-azacytidine (5-aza) treatments, which block the addition of methyl groups to cytosine by Dcm (23, 90).

**TABLE 2:** Summary of candidate genes for regulation by local Dam and Dcm methylation based on available literature, compared with our IPOD-HR, RNAP-ChIP, and RNA-seq results^*a*^

Gene	experiment	Transcription effect	IPOD-HR	RNAP-ChIP	RNA-seq		Citation
					log2FC	q-val	
Dam targets							
*dnaA*	*dam* deletion	–		X	−0.1	1	(87)
		(β-gal)					
	*lexA* deletion	–					
		(β-gal)					
*flu*	Antagonism between methylation and OxyR binding in *flu* promoter (EMSA digestion assay)	–	?	?	−2.42	1	(18, 88)
	OxyR protecting methylation site showed repression (β-gal assay)	(β-gal)					
*glnS*	*dam* deletion	+		X	+1.08	2.84E-13	(86)
	Dam site mutation	(β-gal)					
*ppiA*	Identification of unmethylated	N/A	X	X	−0.22	0.02	(89)
	Dam site						
*mtlA*	Identification of unmethylated	N/A			−0.19	0.03	(89)
	Dam site						
*mtlD*	Identification of unmethylated	N/A			+0.00	0.11	(89)
	Dam site						
*mtlR*	Identification of unmethylated	N/A		X	−0.15	0.76	(89)
	Dam site						
Dcm Targets							
*sugE*	5-aza	+		X	+0.98	1.59E-04	(23, 90)
	*dcm* deletion	(RT-qPCR)					
*rpoS*	*dcm* deletion	+			−0.06	0.78	(22)
		(microarray)					
*osmE*	5-aza	–		X	−0.78	0.02	(90)
		(microarray; RT-qPCR)					
*recN*	5-aza	+			+0.13	0.16	(90)
		(microarray; RT-qPCR)					
*dinD*	5-aza	+			+0.42	1	(90)
		(microarray; RT-qPCR)					
*rsmI*	5-aza	+		X	+1.54	0.04	(90)
		(microarray; RT-qPCR)					
*dinB*	5-aza	+		X	+0.1	1	(90)
		(microarray; RT-qPCR)					
*rmuC*	5-aza	+			+0.5	1	(90)
		(microarray; RT-qPCR)					
*dinG*	5-aza	+			−0.08	1	(90)
		(microarray; RT-qPCR)					
*yqeC*	5-aza	–			-inf	1	(90)
		(microarray; RT-qPCR)					
*recA*	5-aza	+		X	+0.02	1	(90)
		(microarray; RT-qPCR)					

^*a*^ The “Experiment” column briefly describes the conditions in the citation that perturb methylation, and “Transcription effect” refers to the directionality of the change in expression (and methodology used to measure expression change) for the “Gene” when methylation is lost as reported in the citation. “X” indicates an observable change in occupancy pattern for the respective mutant relative to wild-type, and “?” indicates a noisy signal at that locus which precludes interpretation. RNA-seq results are also reported with respect to mutant versus wild-type. “+” and “–” in the “Transcription effect” column indicate that expression of the gene under consideration increased or decreased, respectively, upon application of the noted intervention. “N/A” indicates that expression of the target gene was not measured in the indicated study.

**Fig 5:**
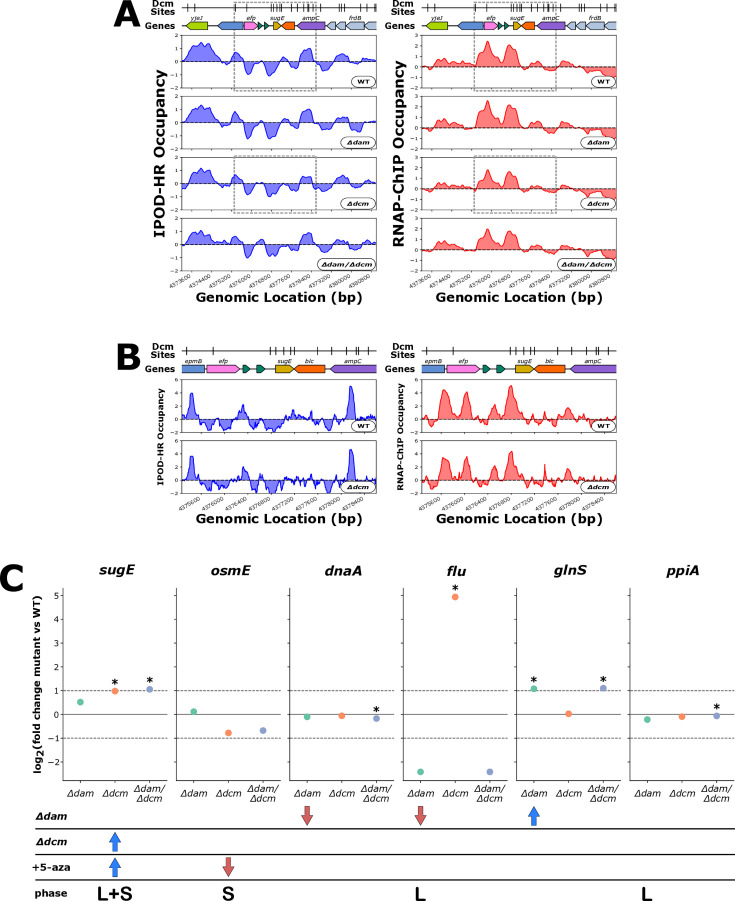
(**A**) Genomic context of *sugE* showing 512 bp rolling mean of IPOD-HR (blue occupancy trace) or RNAP-ChIP (red occupancy trace) robust z-scores. Genes are differentially colored based on their membership to functional gene clusters. The dashed box designates the locus which is shown in panel B. (**B**) Genomic locus of *sugE* showing IPOD-HR (blue occupancy trace) or RNAP-ChIP (red occupancy trace) robust z-scores. (**C**) Mutant versus wild-type change in expression, where the red and blue arrows in the table rows indicate the reported direction of change in expression based on the indicated experiment from curated literature for *sugE* (23, 90), *osmE* (90), *dnaA* (87), *flu* (18, 88), *glnS* (86), and *ppiA* (89). “L” in the growth phase table row stands for “logarithmic,” whereas “S” stands for “stationary” and “L + S” indicates expression data from both logarithic and stationary phase samples. Asterisks indicate statistical significance with a q-value less than 0.05 as calculated by Rockhopper.

Militello *et al*., 2014 (23) identified that in both *dcm* deletion strain and in WT cells subjected to 5-azacytidine treatment, *sugE* is derepressed – our results agree with this finding, as despite a small *Δdcm*-dependent decrease in RNAP-ChIP occupancy at the *sugE* promoter (Fig. 5A and B), there is still an increase in *sugE* transcript levels in our *Δdcm* genotypes (Fig. 5C). While there are several Dcm sites located in the sugE open reading frame, the local IPOD-HR and RNAP-ChIP occupancy signals are generally not altered in our *dcm* deletions. The greatest change in the IPOD-HR signal local to *sugE* is at the stop codon and is of a magnitude that is not likely to indicate biological significance. Thus, we conclude that any relation between Dcm activity and *sugE* expression does not appear to result from occupancy changes dependent on local methylation state, but is instead likely to be an indirect regulatory effect.

In a similar study, Militello et al. 2016 (90) found that 5-azacytidine treatment increases transcript levels of *recN*, *dinD*, *dinG*, *rsmI*, *dinB*, *rmuC*, and *recA* and decreases transcript levels of *osmE* and *yqeC*. Our *dcm* deletion results are consistent with these findings (in terms of the sign of the log fold change upon *dcm* deletion) for all of the genes showing increased transcript levels in (90) except for *dinG,* which we find to drop in expression in our *Δdcm* strain, albeit not significantly (Table 2). For *osmE* and *yqeC*, our *Δdcm* strain shows repression in agreement with the 5-aza study, but *yqeC* is not expressed in any of our strains under our conditions. Therefore, our *dcm* deletion genotype mostly recapitulates the expression changes observed from 5-aza treatment in Militello et al., 2016 (90) except for in the case of *dinG* where we report an opposing impact on gene expression.

**Fig 6:**
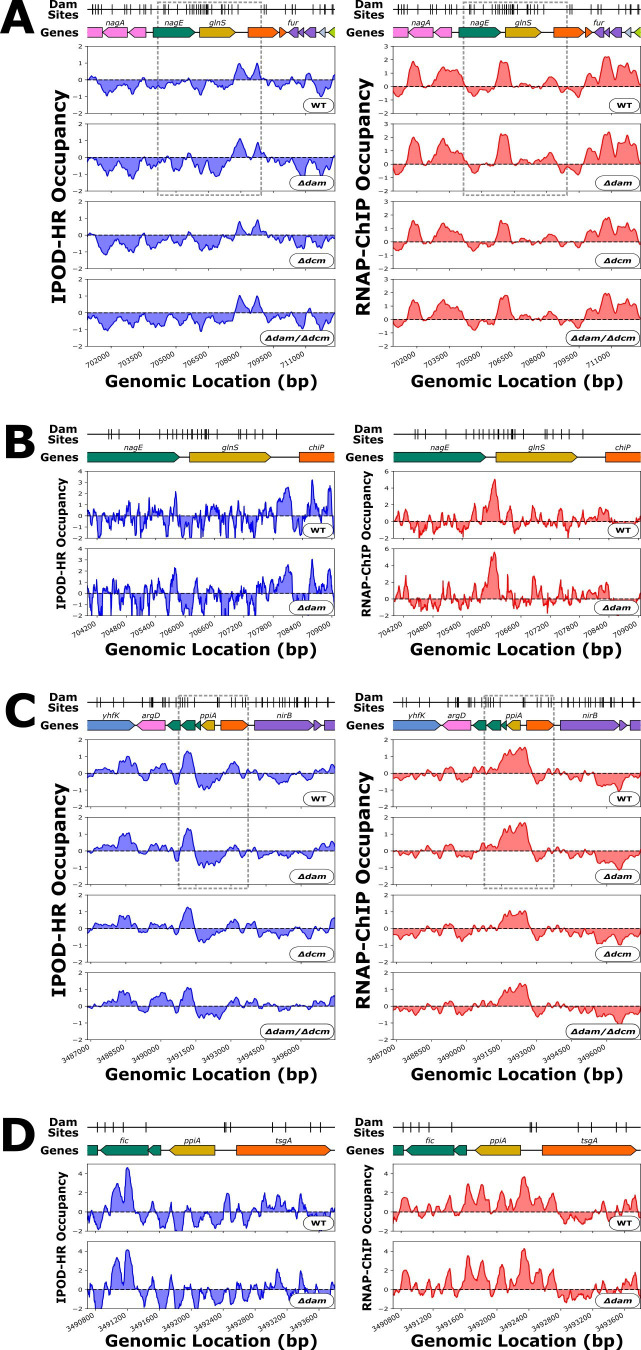
(**A**) Genomic context of *glnS* showing 512 bp rolling mean of IPOD-HR (blue occupancy trace) or RNAP-ChIP (red occupancy trace) robust z-scores. Genes are differentially colored based on their membership to functional gene clusters. The dashed box designates the locus which is shown in panel B. (**B**) Genomic locus of *glnS* showing IPOD-HR (blue occupancy trace) or RNAP-ChIP (red occupancy trace) robust z-scores. (**C**) Genomic context of *ppiA* showing 512 bp rolling mean of IPOD-HR (blue occupancy trace) or RNAP-ChIP (red occupancy trace) robust z-scores. Genes are differentially colored based on their membership to functional gene clusters. The dashed box designates the locus which is shown in panel D. (**D**) Genomic locus of *ppiA* showing IPOD-HR (blue occupancy trace) or RNAP-ChIP (red occupancy trace) robust z-scores.

Several similar data sets have been obtained to study specific instances of regulation of transcription by Dam. Braun and Wright 1986 (87) conducted an *in vivo* β-Galactosidase activity assay and S1 nuclease mapping in addition to *in vitro* transcription run-off experiments, which all supported that loss of Dam methylation in the *dnaA* promoter leads to repression of *dnaA*. We found repression of *dnaA* in our *Δdam* genotypes, but we could not identify any protein occupancy change proximal to the *dnaA* promoter between our wild-type and *Δdam* strains in our IPOD-HR results, possibly due to the competition between DnaA and SeqA for binding to this region (91, 92) (as IPOD-HR would not distinguish between the two factors) or the fact that all of our experiments are ensemble averages over actively growing populations. Correnti et al., 2002 (88) and Wallecha et al*.* 2002 (18) both investigated antagonism between OxyR and Dam methylation in the promoter of *flu,* where the loss of methylation led to OxyR binding, which led to *flu* repression. Our data support that *dam* deletion is associated with *flu* repression, but further assessment of OxyR-methylation antagonism is made difficult due to noisy IPOD-HR and RNAP-ChIP signal at the *flu* promoter, which may be due to issues in data processing and quantitation as a result of repetitive sequence from the transposable element present in that region. Plumbridge and Söll 1987 (86) performed *in vivo* β-Galactosidase activity assays, which showed that *dam* deletion as well as mutation of Dam sites in the promoter leads to derepression of *glnS*. Here, we find, consistently, that *glnS* is more strongly expressed in our *Δdam* strains. It is, however, notable that there is a marginal increase in RNAP-ChIP occupancy at the *glnS* promoter in only the *Δdam* single deletion mutant, and IPOD-HR occupancy at this locus is roughly static across our genotypes (Fig. 6A and B).

Hale et al. 2004 (89) identified under various growth conditions the Dam sites that remain specifically unmethylated throughout the cell cycle. Of the genes reported to be proximal to these stably unmethylated Dam sites, we focus here on *ppiA,* as in our data there are IPOD-HR occupancy changes at upstream Dam sites when *dam* is deleted (Fig. 6C and D). The *Δdam*-dependent loss in non-RNAP protein occupancy directly at Dam sites in a promoter region, as seen with *ppiA,* is precisely what we would expect to observe in a case of methylation-protein antagonism. However, it is not clear whether the *Δdam*-dependent occupancy change has an impact on transcription, as there is a slight increase in RNAP-ChIP occupancy at the promoter in *Δdam* alongside a minimal—although statistically significant—decrease in *ppiA* transcript levels.

Integrating all previous compatible data that we could identify (as detailed above), our RNA-seq data show the same directions of expression changes as prior studies for 13/15 cases (although the changes were not always statistically significant). We did not, however, observe evidence for local changes in protein occupancy at the promoters for most of those genes in response to methyltransferase deletion (with *ppiA* being the primary exception), indicating that either the identities of bound proteins change but the existence of binding does not, that the regulation due to the targeted methyltransferase is indirect, or that we are not sensitive in our assay to any changes that might occur.

### *dam* deletion mutants show loss of motility and downregulation of *flhDC*

One of the genomic loci with a dense clustering of 7 Dam sites was identified as the transcription start site of *flgN*, which encodes for a chaperone involved in cellular export of flagellum components (93, 94). Our RNA-seq results show that *flgN* is downregulated in *dam* deletion mutants (Rockhopper mutants versus wild-type log_2_FC / q-values of *flgN*: −1.2/9.0 × 10^−16^ for *Δdam* vs. WT, −0.20/0.017 for *Δdcm* vs. WT, −0.97/2.9 × 10^−21^ for *Δdam/Δdcm* vs. WT). Additionally, there are mutant-specific IPOD-HR occupancy changes in the *flgN* promoter proximal to the Dam site cluster (Fig. S6). To our knowledge, there is no experimental evidence for what regulatory proteins might act on the promoter immediately upstream of *flgN*, but two distal upstream promoters that impact *flgN* expression have previously been found to be occupied by CsgD and FlhDC (95–97). Although *csgD* transcript levels remain near-zero for all our genotypes, *flhC* and *flhD* transcript levels are decreased in our *Δdam* strains, and FlhDC was previously shown to activate expression of *flgN* (97), thus providing a plausible path of information flow from *dam* deletion to decreased *flgN* transcription.

**Fig 7:**
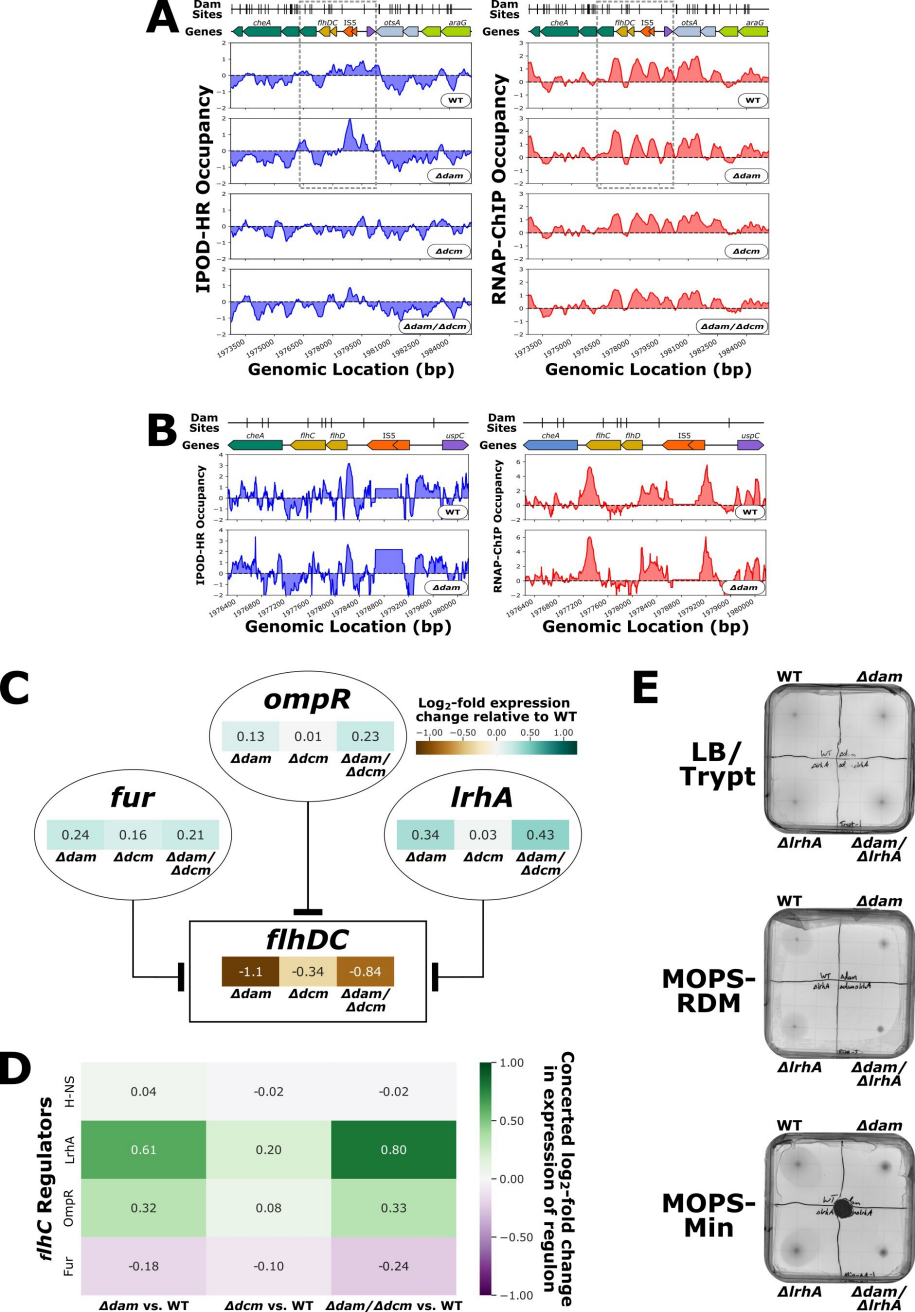
**(A**) Genomic context of *flhDC* showing 512 bp rolling mean of IPOD-HR (blue occupancy trace) or RNAP-ChIP (red occupancy trace) robust z-scores. Genes are differentially colored based on their membership to functional gene clusters. The dashed box designates the locus which is shown in panel B. (**B**) Immediate surroundings of *flhDC* (boxed region from panel A) showing IPOD-HR (blue occupancy trace) or RNAP-ChIP (red occupancy trace) robust z-scores. (**C**) Regulatory diagram including mutant versus wild-type log-fold expression change for the selected subset of regulators of *flhDC*. The lines ending in flat bar arrowheads indicate repression. (**D**) Concerted log_2_-fold change in expression of the regulon of a selected subset of the regulators of *flhDC*. (**E**) Representative motility assay plates for LB/Tryptone, MOPS-RDM, and MOPS-Minimal conditions. The black circle in the center of the MOPS-Minimal plate is a filter disc soaked in aspartic acid which acts as a chemoattractant.

We next analyzed our data sets for information on the expression and protein occupancy of *flhDC*. Although there is protein occupancy in the *flhDC* promoter, the occupancy pattern there appears to differ only marginally based on genotype, suggesting no major changes in the binding of regulatory factors upstream of *flhDC* (Fig. 7A and B). We examined the expression levels of the large set of known *flhDC* regulators across our genotypes to infer what regulators may be responsible for the occupancy signal in the *flhDC* promoter (Fig. 7C). Although this is an indirect inference, we also observed the mutant versus wild-type expression change across the regulons respective to each *flhDC* regulator to check which regulators of *flhDC* likely changed substantially in activity in each mutant (which would be indicated by the presence of changes in expression across the regulon of a factor that were coherent in sign with consideration of the effect of that regulator). The mutant versus wild-type log-fold change in expression of each regulon component was made positive if the change in expression matched the reported mode of regulation for the regulator-target pair or negative if the expression change opposed the annotated regulatory mode. The mean of these sign-changed log-fold expression changes within each regulon was then averaged to calculate the concerted log-fold change in expression of a regulon for each regulator of *flhDC* (Fig. 7D; Fig. S7), with a more positive change indicating stronger evidence for systematic changes throughout the regulon of a given upstream factor in line with its known regulatory effects.

Of the known regulators of *flhDC,* LrhA stands out as having particularly high regulatory coherence scores in both of our strains lacking *dam* (Fig. 7D). LrhA is a repressor of *flhDC* transcription, and *lrhA* expression is increased in *Δdam* strains, which is congruent with *flhDC* downregulation in *Δdam* genotypes (98) and with the broader expression changes in the LrhA regulon in *dam* deletion mutants relative to WT. Fur and OmpR are also repressors of *flhDC* (99, 100) with increased expression in *Δdam* strains, but there is only a weaker degree of concerted log-fold change in OmpR regulon expression for *dam* deletion mutants relative to wild-type, and the Fur regulon does not show signs of strong concerted expression changes (Fig. 7C and D).

To characterize how loss of DNA methylation impacts flagellar motility, we performed swimming motility assays with wild-type, *Δdam, Δdcm*, and *Δdam/Δdcm* strains. To further characterize how the loss of LrhA regulation of *flhDC* impacts motility relative to the motility impact associated with *dam* deletion, we additionally tested *ΔlrhA* and *Δdam/ΔlrhA* strains for swimming motility. Although previous swimming motility assays have utilized tryptone-based motility plates (70), the use of MOPS-RDM in our IPOD-HR and RNA-seq procedures motivated us to develop MOPS-RDM and MOPS-glucose motility plates. Across all media types used for the motility assays, *dam* deletion mutants consistently demonstrated a substantial loss of motility as observed by the swimming distance of cells over time (Fig. 7E), as would be expected based on our RNA-seq results. *lrhA* deletion mutants appear to have a small loss of motility in all tested media conditions, and the *Δdam/ΔlrhA* strain displays slightly less swimming motility than the *Δdam* strain. Although we observe decreased LrhA expression and regulatory activity in *Δdam* strains, the swimming motility phenotypes (and particularly the lack of apparent epistasis between *dam* and *lrhA* deletions) do not provide further insight into the nature of the relationship between LrhA and Dam in regulation of motility; one thing that is clearly apparent is that the loss of motility of *Δdam* mutants cannot be attributed solely to the increase in LrhA activity, since the phenotype persists in the *Δdam/ΔlrhA* double knockout.

## DISCUSSION

Based on previous observations of depletion of Dam methylation sites in extended, transcriptionally silent regions of high protein occupancy in the *E. coli* genome, we hypothesized that Dam methylation might play a global role in regulating the spread of NAP occupancy to control where EPODs occur, by inhibiting NAP occupancy in regions with relatively high Dam site densities. Contrary to our initial hypothesis, our results indicate that DNA methylation state (at least that arising from the native *E. coli* K12 DNA methyltransferases) does not substantially impact the global pattern of protein occupancy and EPOD formation at methylation sites. Thus, we observe that the genome-wide association between Dam sites and EPODs (46) is not causal but likely reflects other evolutionary constraints acting on long-term versus newly acquired genomic regions. Many EPODs in *E. coli* K-12 MG1655 have been observed to be associated with prophages and transposable elements (47), which were likely acquired more recently and may have experienced less selective pressure, over less time, for containing Dam sites relative to more native regions of the genome (77, 78, 101). Although our data did not demonstrate a global pattern of DNA methylation state regulating local protein occupancy, there may still be a small number of methylation sites where antagonism exists between DNA methyltransferases and regulatory proteins. We also note that our tested conditions were limited to standard growth in rich medium (MOPS-RDM), and thus, it is possible that changes in occupancy that would occur under other growth conditions are missed (although a global condition-independent effect on EPOD positioning can certainly be ruled out). For example, our cells were harvested at the exponential phase, but DNA methylation—and Dcm methylation in particular—appears to have a more biologically significant impact on gene expression during the stationary phase (22, 36, 72, 102). Therefore, the possibility remains that DNA methylation may have a more pronounced impact on chromatin structure during the stationary phase, which may be associated with changes in gene expression over the bacterial growth cycle. Another limitation of note is that deletion of methyltransferases is not expected to produce stably hemimethylated Dam sites, which might more specifically interact with some DNA-binding proteins relative to unmethylated or fully methylated sites (as is the case with SeqA) (103–105), and thus, we might miss changes in occupancy arising specifically from the presence of hemimethylated regions. Furthermore, our exploration of the mechanistic link between loss of DNA methyltransferases and changes in transcript abundance is confined to the level of transcription initiation. The possibility of other DNA methylation impacting other regulatory stages of transcription such as elongation and termination is not explored in this study.

Nevertheless, we did find that some protein occupancy changes caused by *dam* deletion are associated with loci featuring a dense clustering of methylation sites—such sites do show a decrease in total protein occupancy and an increase in RNA polymerase occupancy for *dam* deletion strains. Genomic regions with multiple proximal Dam sites have been previously identified as a potential regulatory element due to the poor processivity of Dam over such regions, resulting in hemimethylated sites (1, 106, 107). Stably hemi- or un-methylated Dam sites are rare relative to fully methylated sites, and hence, they have been predicted to form specific associations with DNA-binding proteins such as SeqA (1, 28). However, loss of methylation at the dense Dam site clusters we identified does not appear to generally result in differential regulation of known local transcripts. We thus conclude that the *Δdam*-dependent changes in protein occupancy associated with dense clusters of Dam sites are primarily driven by the increased presence of RNA polymerase, which is not transcriptionally active under our conditions, possibly due to increased promoter binding/transcriptional initiation without promoter clearance.

We observed the presence of an RNAP-ChIP peak proximal to all of our observations of high-density Dam site clusters at the ends of gene bodies, and we speculated that this RNAP-ChIP peak results either from direct recruitment of RNAP or stalling of RNAP at this site during Dam-associated mismatch repair (28, 30, 85). Our samples for RNAP-ChIP and IPOD-HR are treated with rifampicin before crosslinking, and rifampicin inhibits promoter clearance of RNA polymerase (46, 83, 84). Thus, we hypothesized that RNA polymerase recruited to an upstream promoter could read through a gene, get stalled at the Dam site cluster, and then be prevented from dissociating from the DNA by rifampicin until formaldehyde crosslinking. Although our read-end analysis did not support the presence of DNA damage at these loci, any potential direct signature of accumulations of strand breaks could easily have been masked by our sample workup, and we still suspect that stalling of the RNAP by DNA repair machinery is possible due to other aspects of dysregulated replication in *Δdam* strains such as asynchronous replication initiation and DNA base mismatches (79, 108). Contradicting the alternative scenario in which RNAP might be directly recruited to these Dam site clusters, we found a lack of changes in local transcript levels; however, it is still possible that RNAP could be recruited for the transcription of sRNAs (109), but this transcription is not active (e.g., due to the lack of promoter clearance) under our conditions or that we were not able to detect these small transcripts.

To characterize another example of a dense Dam site cluster that shows substantial changes in protein occupancy, we considered a cluster occurring near the flagellar chaperone gene *flgN*. Our investigation was motivated by the presence of a dense Dam site cluster at the promoter of *flgN* as well as *Δdam*-associated downregulation of flagellum synthesis genes, which led us to characterize the regulatory network governing flagellar synthesis and swimming motility in our methyltransferase mutants. We focused on the master regulator FlhDC since it is a regulator of *flgN*, and *flhDC* expression is decreased in *Δdam* strains. To explore a possible causal relationship between DNA methylation and protein occupancy leading to *Δdam*-associated changes in the regulatory network governing flagellum synthesis, we expanded our investigation to include regulators of *flhDC*. Based on the analysis of expression changes in the regulons of each *flhDC* regulator, we identified LrhA as the most likely regulator of *flhDC* to be differentially regulating its targets in response to methyltransferase deletion, but characterization of the swimming motility for *lrhA* and *dam* deletion strains did not reveal a clear regulatory relationship between LrhA and *dam* deletion. We also note that there are multiple transposable elements that may be incorporated upstream of *flhDC*, and the presence of these transposable elements has been shown to impact *flhDC* expression and flagellum-based motility (77, 110); we did not, however, observe any consistent pattern in our samples of changes in transposable elements around the *flhDC* promoter (data not shown). Although the full nature of the relationship between DNA methylation and motility remains elusive, here, we have demonstrated that loss of Dam methylation is associated with a substantial loss of swimming-based motility.

Although our observations suggest that loss of *lrhA* leads to a decrease in swimming motility (98), we transduced an insertionally inactivated *lrhA* into the MG1655 background and found that functional loss of *lrhA* leads to an increase in swimming motility (98). Our laboratory strain of MG1655 has an IS1 insertion in the coding region of *dgcJ* (47, 48), which is a gene encoding for a diguanylate cyclase that has been associated with the regulation of swimming motility (111). It is thus possible that an epistatic interaction between *lrhA* and *dgcJ* explains the discrepancy in swimming motility phenotype resulting from functional loss of *lrhA* between our findings and those of Lehnen et al. 2002 (98), particularly given the importance of cyclic di-GMP for regulating flagellar motility (112).

In globally surveying the impact of the loss of DNA methylation on gene expression and protein occupancy in *E. coli* K-12 MG1655, our results indicate that although the loss of *dam* and/or *dcm* leads to statistically and biologically significant changes in gene expression associated with observable phenotypes—such as loss of swimming motility—these changes appear to result primarily from global physiological effects of *dam* or *dcm* loss rather than being due to transcriptional regulatory consequences of losing local DNA methylation signal. Our observations of protein occupancy change at methylation sites are primarily at loci with exceptionally dense clustering of Dam sites, where we observe an increase in RNAP occupancy, but we find this pattern to be of no consequence to local transcriptional output. We thus conclude that DNA methylation is not a biologically significant factor in local gene expression or global chromatin structure for *E. coli* K-12 MG1655 under our tested conditions. Future studies that aim to address the question of whether there is any regulatory interplay between NAP or transcription factor occupancy, and DNA methylation in MG1655 would be well-served by either testing a wider range of growth conditions or employing site-specific perturbation of methylation status without altering DNA sequence (e.g., with a tethered methyltransferase or demethylase) (113, 114).

## SUPPLEMENTAL MATERIAL

Supplemental figures legends jb.00540-24-s0001.pdf Legends for Fig. S1 to S8.

Figure S1 jb.00540-24-s0002.tif Gene set enrichment analysis for RNA-seq data across the indicated genotypes (relative to wild type).

Figure S2 jb.00540-24-s0003.tif Distribution of changes in mean IPOD-HR or RNAP-ChIP occupancy scores.

Figure S3 jb.00540-24-s0004.tif Symmetrized Overlap Distances calculated.

Figure S4 jb.00540-24-s0005.tif Genomic context of (A) selB, (B) prpE, and (C) recBD.

Figure S5 jb.00540-24-s0006.tif Normalized read ends.

Figure S6 jb.00540-24-s0007.tif (A) Genomic context of flgN (B) Genomic locus of flgN.

Figure S7 jb.00540-24-s0008.tif Heatmap representing the expression change in the regulon of regulators of flhC.

Figure S8 jb.00540-24-s0009.tif Semilog plots.

ASM does not own the copyrights to Supplemental Material that may be linked to, or accessed through, an article. The authors have granted ASM a non-exclusive, world-wide license to publish the Supplemental Material files. Please contact the corresponding author directly for reuse.

## References

[1] Marinus MG, Løbner-Olesen A. 2014. DNA methylation. EcoSal Plus 6:10. doi:10.1128/ecosalplus.ESP-0003-2013 PMC4231299 26442938

[2] Wilson GG, Murray NE. 1991. Restriction and modification systems. Annu Rev Genet 25:585–627. doi:10.1146/annurev.ge.25.120191.003101 1812816

[3] Labrie SJ, Samson JE, Moineau S. 2010. Bacteriophage resistance mechanisms. Nat Rev Microbiol 8:317–327. doi:10.1038/nrmicro2315 20348932

[4] Herman GE, Modrich P. 1982. Escherichia coli dam methylase. physical and catalytic properties of the homogeneous enzyme. J Biol Chem 257:2605–2612. doi:10.1016/S0021-9258(18)34967-6 7037767

[5] Wu JC, Santi DV. 1987. Kinetic and catalytic mechanism of HhaI methyltransferase. J Biol Chem 262:4778–4786. doi:10.1016/S0021-9258(18)61263-3 3558369

[6] Balbontín R, Rowley G, Pucciarelli MG, López-Garrido J, Wormstone Y, Lucchini S, García-Del Portillo F, Hinton JCD, Casadesús J. 2006. DNA adenine methylation regulates virulence gene expression in Salmonella enterica serovar Typhimurium. J Bacteriol 188:8160–8168. doi:10.1128/JB.00847-06 16997949 PMC1698197

[7] García-Del Portillo F, Pucciarelli MG, Casadesús J. 1999. DNA adenine methylase mutants of Salmonella typhimurium show defects in protein secretion, cell invasion, and M cell cytotoxicity. Proc Natl Acad Sci USA 96:11578–11583. doi:10.1073/pnas.96.20.11578 10500219 PMC18076

[8] Prieto AI, Ramos-Morales F, Casadesús J. 2006. Repair of DNA damage induced by bile salts in Salmonella enterica. Genetics 174:575–584. doi:10.1534/genetics.106.060889 16888329 PMC1602091

[9] Carter MQ, Pham A, Huynh S, Parker CT, Miller A, He X, Hu B, Chain PSG. 2021. DNA adenine methylase, not the PstI restriction-modification system, regulates virulence gene expression in Shiga toxin-producing Escherichia coli. Food Microbiol 96:103722. doi:10.1016/j.fm.2020.103722 33494894

[10] Heithoff DM, Sinsheimer RL, Low DA, Mahan MJ. 1999. An essential role for DNA adenine methylation in bacterial virulence. Science 284:967–970. doi:10.1126/science.284.5416.967 10320378

[11] Gonzalez D, Kozdon JB, McAdams HH, Shapiro L, Collier J. 2014. The functions of DNA methylation by CcrM in Caulobacter crescentus: a global approach. Nucleic Acids Res 42:3720–3735. doi:10.1093/nar/gkt1352 24398711 PMC3973325

[12] Marczynski GT, Shapiro L. 2002. Control of chromosome replication in Caulobacter crescentus. Annu Rev Microbiol 56:625–656. doi:10.1146/annurev.micro.56.012302.161103 12142494

[13] Collier J. 2009. Epigenetic regulation of the bacterial cell cycle. Curr Opin Microbiol 12:722–729. doi:10.1016/j.mib.2009.08.005 19783470

[14] Wang MX, Church GM. 1992. A whole genome approach to in vivo DNA-protein interactions in E. coli. Nature 360:606–610. doi:10.1038/360606a0 1334233

[15] Tavazoie S, Church GM. 1998. Quantitative whole-genome analysis of DNA-protein interactions by in vivo methylase protection in E. coli. Nat Biotechnol 16:566–571. doi:10.1038/nbt0698-566 9624689

[16] Blyn LB, Braaten BA, Low DA. 1990. Regulation of pap pilin phase variation by a mechanism involving differential dam methylation states. EMBO J 9:4045–4054. doi:10.1002/j.1460-2075.1990.tb07626.x 2147413 PMC552177

[17] Zamora M, Ziegler CA, Freddolino PL, Wolfe AJ. 2020. A thermosensitive, phase-variable epigenetic switch: pap revisited. Microbiol Mol Biol Rev 84:e00030–17. doi:10.1128/mmbr.00030-17 PMC7392537 32727743

[18] Wallecha A, Munster V, Correnti J, Chan T, van der Woude M. 2002. Dam- and OxyR-dependent phase variation of agn43: essential elements and evidence for a new role of DNA methylation. J Bacteriol 184:3338–3347. doi:10.1128/JB.184.12.3338-3347.2002 12029051 PMC135096

[19] Brunet YR, Bernard CS, Cascales E. 2020. Fur-dam regulatory interplay at an internal promoter of the enteroaggregative Escherichia coli type VI secretion sci1 gene cluster. J Bacteriol 202:e00075-20. doi:10.1128/JB.00075-20 32152218 PMC7186456

[20] Seshasayee ASN. 2007. An assessment of the role of DNA adenine methyltransferase on gene expression regulation in E coli. PLoS One 2:e273. doi:10.1371/journal.pone.0000273 17342207 PMC1804101

[21] Løbner-Olesen A, Marinus MG, Hansen FG. 2003. Role of SeqA and Dam in Escherichia coli gene expression: a global/microarray analysis. Proc Natl Acad Sci USA 100:4672–4677. doi:10.1073/pnas.0538053100 12682301 PMC153614

[22] Kahramanoglou C, Prieto AI, Khedkar S, Haase B, Gupta A, Benes V, Fraser GM, Luscombe NM, Seshasayee ASN. 2012. Genomics of DNA cytosine methylation in Escherichia coli reveals its role in stationary phase transcription. Nat Commun 3:886. doi:10.1038/ncomms1878 22673913

[23] Militello KT, Mandarano AH, Varechtchouk O, Simon RD. 2014. Cytosine DNA methylation influences drug resistance in Escherichia coli through increased sugE expression. FEMS Microbiol Lett 350:100–106. doi:10.1111/1574-6968.12299 24164619

[24] Geier GE, Modrich P. 1979. Recognition sequence of the dam methylase of Escherichia coli K12 and mode of cleavage of Dpn I endonuclease. J Biol Chem 254:1408–1413. 368070

[25] Murphy J, Mahony J, Ainsworth S, Nauta A, van Sinderen D. 2013. Bacteriophage orphan DNA methyltransferases: insights from their bacterial origin, function, and occurrence. Appl Environ Microbiol 79:7547–7555. doi:10.1128/AEM.02229-13 24123737 PMC3837797

[26] Messer W, Bellekes U, Lother H. 1985. Effect of dam methylation on the activity of the E. coli replication origin, oriC. EMBO J 4:1327–1332. doi:10.1002/j.1460-2075.1985.tb03780.x 3891330 PMC554344

[27] Yamaki H, Ohtsubo E, Nagai K, Maeda Y. 1988. The oriC unwinding by dam methylation in Escherichia coli. Nucleic Acids Res 16:5067–5073. doi:10.1093/nar/16.11.5067 3290846 PMC336717

[28] Raghunathan N, Goswami S, Leela JK, Pandiyan A, Gowrishankar J. 2019. A new role for Escherichia coli Dam DNA methylase in prevention of aberrant chromosomal replication. Nucleic Acids Res 47:5698–5711. doi:10.1093/nar/gkz242 30957852 PMC6582345

[29] Urig S, Gowher H, Hermann A, Beck C, Fatemi M, Humeny A, Jeltsch A. 2002. The Escherichia coli dam DNA methyltransferase modifies DNA in a highly processive reaction. J Mol Biol 319:1085–1096. doi:10.1016/S0022-2836(02)00371-6 12079349

[30] Pukkila PJ, Peterson J, Herman G, Modrich P, Meselson M. 1983. Effects of high levels of DNA adenine methylation on methyl-directed mismatch repair in Escherichia coli. Genetics 104:571–582. doi:10.1093/genetics/104.4.571 6225697 PMC1202127

[31] Glickman BW, Radman M. 1980. Escherichia coli mutator mutants deficient in methylation-instructed DNA mismatch correction. Proc Natl Acad Sci USA 77:1063–1067. doi:10.1073/pnas.77.2.1063 6987663 PMC348424

[32] Carraway M, Youderian P, Marinus MG. 1987. Spontaneous mutations occur near dam recognition sites in a dam- Escherichia coli host. Genetics 116:343–347. doi:10.1093/genetics/116.3.343 3301526 PMC1203145

[33] Hanck T, Schmidt S, Fritz H-J. 1993. Sequence-specific and mechanism-based crosslinking of Dcm DNA cytosine-C5 methyltransferase of E. coli K-12 to synthetic oligonucleotides containing 5-fluoro-2’-deoxycytidine. Nucleic Acids Res 21:303–309. doi:10.1093/nar/21.2.303 8441638 PMC309107

[34] Russell DW, Hirata RK. 1989. The detection of extremely rare DNA modifications. Methylation in dam- and hsd- Escherichia coli strains. J Biol Chem 264:10787–10794. 2659593

[35] Gómez-Eichelmann MC, Ramírez-Santos J. 1993. Methylated cytosine at Dcm (CCATGG) sites in Escherichia coli: possible function and evolutionary implications. J Mol Evol 37:11–24. doi:10.1007/BF00170457 8360914

[36] Militello KT, Finnerty-Haggerty L, Kambhampati O, Huss R, Knapp R. 2020. DNA cytosine methyltransferase enhances viability during prolonged stationary phase in Escherichia coli. FEMS Microbiol Lett 367:fnaa166. doi:10.1093/femsle/fnaa166 33045036

[37] Hennecke F, Kolmar H, Bründl K, Fritz H-J. 1991. The vsr gene product of E. coli K-12 is a strand- and sequence-specific DNA mismatch endonuclease. Nature 353:776–778. doi:10.1038/353776a0 1944537

[38] Oshima T, Wada C, Kawagoe Y, Ara T, Maeda M, Masuda Y, Hiraga S, Mori H. 2002. Genome-wide analysis of deoxyadenosine methyltransferase-mediated control of gene expression in Escherichia coli. Mol Microbiol 45:673–695. doi:10.1046/j.1365-2958.2002.03037.x 12139615

[39] Polaczek P, Kwan K, Campbell JL. 1998. GATC motifs may alter the conformation of DNA depending on sequence context and N6-adenine methylation status: possible implications for DNA-protein recognition. Mol Gen Genet 258:488–493. doi:10.1007/s004380050759 9669330

[40] Dillon SC, Dorman CJ. 2010. Bacterial nucleoid-associated proteins, nucleoid structure and gene expression. Nat Rev Microbiol 8:185–195. doi:10.1038/nrmicro2261 20140026

[41] Hołówka J, Zakrzewska-Czerwińska J. 2020. Nucleoid associated proteins: the small organizers that help to cope with stress. Front Microbiol 11:590. doi:10.3389/fmicb.2020.00590 32373086 PMC7177045

[42] Broadbent SE, Davies MR, van der Woude MW. 2010. Phase variation controls expression of Salmonella lipopolysaccharide modification genes by a DNA methylation-dependent mechanism. Mol Microbiol 77:337–353. doi:10.1111/j.1365-2958.2010.07203.x 20487280 PMC2909390

[43] Rao S, Chiu T-P, Kribelbauer JF, Mann RS, Bussemaker HJ, Rohs R. 2018. Systematic prediction of DNA shape changes due to CpG methylation explains epigenetic effects on protein-DNA binding. Epigenetics Chromatin 11:6. doi:10.1186/s13072-018-0174-4 29409522 PMC5800008

[44] Diekmann S. 1987. DNA methylation can enhance or induce DNA curvature. EMBO J 6:4213–4217. doi:10.1002/j.1460-2075.1987.tb02769.x 2832157 PMC553906

[45] Vora T, Hottes AK, Tavazoie S. 2009. Protein occupancy landscape of a bacterial genome. Mol Cell 35:247–253. doi:10.1016/j.molcel.2009.06.035 19647521 PMC2763621

[46] Freddolino PL, Amemiya HM, Goss TJ, Tavazoie S. 2021. Dynamic landscape of protein occupancy across the Escherichia coli chromosome. PLOS Biol 19:e3001306. doi:10.1371/journal.pbio.3001306 34170902 PMC8282354

[47] Amemiya HM, Goss TJ, Nye TM, Hurto RL, Simmons LA, Freddolino PL. 2022. Distinct heterochromatin-like domains promote transcriptional memory and silence parasitic genetic elements in bacteria. EMBO J 41:e108708. doi:10.15252/embj.2021108708 34961960 PMC8804932

[48] Freddolino PL, Amini S, Tavazoie S. 2012. Newly identified genetic variations in common Escherichia coli MG1655 stock cultures. J Bacteriol 194:303–306. doi:10.1128/jb.06087-11 22081388 PMC3256642

[49] Baba T, Ara T, Hasegawa M, Takai Y, Okumura Y, Baba M, Datsenko KA, Tomita M, Wanner BL, Mori H. 2006. Construction of Escherichia coli K-12 in-frame, single-gene knockout mutants: the Keio collection. Mol Syst Biol 2:2006.0008. doi:10.1038/msb4100050 PMC1681482 16738554

[50] Thomason LC, Costantino N, Court DL. 2007. E. coli genome manipulation by P1 transduction. Curr Protoc Mol Biol Chapter 1:1. doi:10.1002/0471142727.mb0117s79 18265391

[51] Cherepanov PP, Wackernagel W. 1995. Gene disruption in Escherichia coli: TcR and KmR cassettes with the option of Flp-catalyzed excision of the antibiotic-resistance determinant. Gene 158:9–14. doi:10.1016/0378-1119(95)00193-a 7789817

[52] Neidhardt FC, Bloch PL, Smith DF. 1974. Culture medium for enterobacteria. J Bacteriol 119:736–747. doi:10.1128/jb.119.3.736-747.1974 4604283 PMC245675

[53] AUSUBEL F. 1998. *Escherichia coli,* plasmids, and bacteriophages. In Current Protocols in Molecular Biology

[54] Martin M. 2011. Cutadapt removes adapter sequences from high-throughput sequencing reads. EMBnet j 17:10. doi:10.14806/ej.17.1.200

[55] Bolger AM, Lohse M, Usadel B. 2014. Trimmomatic: a flexible trimmer for Illumina sequence data. Bioinformatics 30:2114–2120. doi:10.1093/bioinformatics/btu170 24695404 PMC4103590

[56] Langmead B, Salzberg SL. 2012. Fast gapped-read alignment with Bowtie 2. Nat Methods 9:357–359. doi:10.1038/nmeth.1923 22388286 PMC3322381

[57] Danecek P, Bonfield JK, Liddle J, Marshall J, Ohan V, Pollard MO, Whitwham A, Keane T, McCarthy SA, Davies RM, Li H. 2021. Twelve years of SAMtools and BCFtools. Gigascience 10:giab008. doi:10.1093/gigascience/giab008 33590861 PMC7931819

[58] Li Q, Brown JB, Huang H, Bickel PJ. 2011. Measuring reproducibility of high-throughput experiments. Ann Appl Stat 5:1752–1779. doi:10.1214/11-AOAS466

[59] Cock PJA, Antao T, Chang JT, Chapman BA, Cox CJ, Dalke A, Friedberg I, Hamelryck T, Kauff F, Wilczynski B, de Hoon MJL. 2009. Biopython: freely available Python tools for computational molecular biology and bioinformatics. Bioinformatics 25:1422–1423. doi:10.1093/bioinformatics/btp163 19304878 PMC2682512

[60] Quinlan AR, Hall IM. 2010. BEDTools: a flexible suite of utilities for comparing genomic features. Bioinformatics 26:841–842. doi:10.1093/bioinformatics/btq033 20110278 PMC2832824

[61] Waskom ML. 2021. Seaborn: statistical data visualization. JOSS 6:3021. doi:10.21105/joss.03021

[62] R Core Team. 2021. R: a language and environment for statistical computing (R foundation for statistical computing)

[63] Wickham H. 2016. Ggplot2: elegant graphics for data analysis. Springer-Verlag New York.

[64] Tjaden B. 2020. A computational system for identifying operons based on RNA-seq data. Methods 176:62–70. doi:10.1016/j.ymeth.2019.03.026 30953757 PMC6776731

[65] Tjaden B. 2015. De novo assembly of bacterial transcriptomes from RNA-seq data. Genome Biol 16:1. doi:10.1186/s13059-014-0572-2 25583448 PMC4316799

[66] McClure R, Balasubramanian D, Sun Y, Bobrovskyy M, Sumby P, Genco CA, Vanderpool CK, Tjaden B. 2013. Computational analysis of bacterial RNA-Seq data. Nucleic Acids Res 41:e140. doi:10.1093/nar/gkt444 23716638 PMC3737546

[67] Hunter JD. 2007. Matplotlib: A 2D graphics environment. Comput Sci Eng 9:90–95. doi:10.1109/MCSE.2007.55

[68] Goodarzi H, Elemento O, Tavazoie S. 2009. Revealing global regulatory perturbations across human cancers. Mol Cell 36:900–911. doi:10.1016/j.molcel.2009.11.016 20005852 PMC2900319

[69] Gama-Castro S, Salgado H, Santos-Zavaleta A, Ledezma-Tejeida D, Muñiz-Rascado L, García-Sotelo JS, Alquicira-Hernández K, Martínez-Flores I, Pannier L, Castro-Mondragón JA, Medina-Rivera A, Solano-Lira H, Bonavides-Martínez C, Pérez-Rueda E, Alquicira-Hernández S, Porrón-Sotelo L, López-Fuentes A, Hernández-Koutoucheva A, Del Moral-Chávez V, Rinaldi F, Collado-Vides J. 2016. RegulonDB version 9.0: high-level integration of gene regulation, coexpression, motif clustering and beyond. Nucleic Acids Res 44:D133–43. doi:10.1093/nar/gkv1156 26527724 PMC4702833

[70] Ha D-G, Kuchma SL, O’Toole GA. 2014. Plate-based assay for swimming motility in Pseudomonas aeruginosa. Methods Mol Biol 1149:59–65. doi:10.1007/978-1-4939-0473-0_7 24818897 PMC9007281

[71] ImageMagick Studio LLC. 2024. ImageMagick

[72] Westphal LL, Sauvey P, Champion MM, Ehrenreich IM, Finkel SE. 2016. Genomewide dam methylation in Escherichia coli during long-term stationary phase. mSystems 1:e00130-16. doi:10.1128/mSystems.00130-16 27981240 PMC5155068

[73] Nobelmann B, Lengeler JW. 1996. Molecular analysis of the gat genes from Escherichia coli and of their roles in galactitol transport and metabolism. J Bacteriol 178:6790–6795. doi:10.1128/jb.178.23.6790-6795.1996 8955298 PMC178577

[74] Dippel R, Boos W. 2005. The maltodextrin system of Escherichia coli: metabolism and transport. J Bacteriol 187:8322–8331. doi:10.1128/JB.187.24.8322-8331.2005 16321936 PMC1316994

[75] Friden P, Newman T, Freundlich M. 1982. Nucleotide sequence of the ilvB promoter-regulatory region: a biosynthetic operon controlled by attenuation and cyclic AMP. Proc Natl Acad Sci USA 79:6156–6160. doi:10.1073/pnas.79.20.6156 6292893 PMC347078

[76] Peterson KR, Wertman KF, Mount DW, Marinus MG. 1985. Viability of Escherichia coli K-12 DNA adenine methylase (dam) mutants requires increased expression of specific genes in the SOS regulon. Mol Gen Genet 201:14–19. doi:10.1007/BF00397979 3932821

[77] Yin JCP, Krebs MP, Reznikoff WS. 1988. Effect of dam methylation on Tn5 transposition. J Mol Biol 199:35–45. doi:10.1016/0022-2836(88)90377-4 2451025

[78] Roberts D, Hoopes BC, McClure WR, Kleckner N. 1985. IS10 transposition is regulated by DNA adenine methylation. Cell 43:117–130. doi:10.1016/0092-8674(85)90017-0 3000598

[79] Robbins-Manke JL, Zdraveski ZZ, Marinus M, Essigmann JM. 2005. Analysis of global gene expression and double-strand-break formation in DNA adenine methyltransferase- and mismatch repair-deficient Escherichia coli. J Bacteriol 187:7027–7037. doi:10.1128/JB.187.20.7027-7037.2005 16199573 PMC1251628

[80] Park PJ. 2009. ChIP-seq: advantages and challenges of a maturing technology. Nat Rev Genet 10:669–680. doi:10.1038/nrg2641 19736561 PMC3191340

[81] Kang S, Lee H, Han JS, Hwang DS. 1999. Interaction of SeqA and Dam methylase on the hemimethylated origin of Escherichia coli chromosomal DNA replication. J Biol Chem 274:11463–11468. doi:10.1074/jbc.274.17.11463 10206949

[82] Marinus MG, Morris NR. 1974. Biological function for 6-methyladenine residues in the DNA of Escherichia coli K12. J Mol Biol 85:309–322. doi:10.1016/0022-2836(74)90366-0 4600143

[83] McClure WR, Cech CL. 1978. On the mechanism of rifampicin inhibition of RNA synthesis. J Biol Chem 253:8949–8956. doi:10.1016/S0021-9258(17)34269-2 363713

[84] Campbell EA, Korzheva N, Mustaev A, Murakami K, Nair S, Goldfarb A, Darst SA. 2001. Structural mechanism for rifampicin inhibition of bacterial RNA polymerase. Cell 104:901–912. doi:10.1016/s0092-8674(01)00286-0 11290327

[85] Wang W, Xu L, Hu L, Chong J, He C, Wang D. 2017. Epigenetic DNA modification N^6^-methyladenine causes site-specific RNA polymerase ii transcriptional pausing. J Am Chem Soc 139:14436–14442. doi:10.1021/jacs.7b06381 28933854 PMC5812728

[86] Plumbridge J, Söll D. 1987. The effect of dam methylation on the expression of glnS in E. coli. Biochimie 69:539–541. doi:10.1016/0300-9084(87)90091-5 2960382

[87] Braun RE, Wright A. 1986. DNA methylation differentially enhances the expression of one of the two E. coli dnaA promoters in vivo and in vitro. Mol Gen Genet 202:246–250. doi:10.1007/BF00331644 3010047

[88] Correnti J, Munster V, Chan T, Woude M van der. 2002. Dam-dependent phase variation of Ag43 in Escherichia coli is altered in a seqA mutant. Mol Microbiol 44:521–532. doi:10.1046/j.1365-2958.2002.02918.x 11972788

[89] Hale WB, van der Woude MW, Low DA. 1994. Analysis of nonmethylated GATC sites in the Escherichia coli chromosome and identification of sites that are differentially methylated in response to environmental stimuli. J Bacteriol 176:3438–3441. doi:10.1128/jb.176.11.3438-3441.1994 8195106 PMC205523

[90] Militello KT, Simon RD, Mandarano AH, DiNatale A, Hennick SM, Lazatin JC, Cantatore S. 2016. 5-azacytidine induces transcriptome changes in Escherichia coli via DNA methylation-dependent and DNA methylation-independent mechanisms. BMC Microbiol 16:130. doi:10.1186/s12866-016-0741-4 27349222 PMC4924334

[91] Taghbalout A, Landoulsi A, Kern R, Yamazoe M, Hiraga S, Holland B, Kohiyama M, Malki A. 2000. Competition between the replication initiator DnaA and the sequestration factor SeqA for binding to the hemimethylated chromosomal origin of E. coli in vitro*.* Genes Cells 5:873–884. doi:10.1046/j.1365-2443.2000.00380.x 11122375

[92] Nievera C, Torgue JJ-C, Grimwade JE, Leonard AC. 2006. SeqA blocking of DnaA-oriC interactions ensures staged assembly of the E. coli pre-RC. Mol Cell 24:581–592. doi:10.1016/j.molcel.2006.09.016 17114060 PMC1939805

[93] Fraser GM, Bennett JC, Hughes C. 1999. Substrate-specific binding of hook-associated proteins by FlgN and FliT, putative chaperones for flagellum assembly. Mol Microbiol 32:569–580. doi:10.1046/j.1365-2958.1999.01372.x 10320579

[94] Bennett JC, Thomas J, Fraser GM, Hughes C. 2001. Substrate complexes and domain organization of the Salmonella flagellar export chaperones FlgN and FliT. Mol Microbiol 39:781–791. doi:10.1046/j.1365-2958.2001.02268.x 11169117 PMC2528293

[95] Dudin O, Geiselmann J, Ogasawara H, Ishihama A, Lacour S. 2014. Repression of flagellar genes in exponential phase by CsgD and CpxR, two crucial modulators of Escherichia coli biofilm formation. J Bacteriol 196:707–715. doi:10.1128/JB.00938-13 24272779 PMC3911157

[96] Stafford GP, Ogi T, Hughes C. 2005. Binding and transcriptional activation of non-flagellar genes by the Escherichia coli flagellar master regulator FlhD2C2. Microbiology (Reading) 151:1779–1788. doi:10.1099/mic.0.27879-0 15941987 PMC2528288

[97] Fitzgerald DM, Bonocora RP, Wade JT. 2014. Comprehensive mapping of the Escherichia coli flagellar regulatory network. PLoS Genet 10:e1004649. doi:10.1371/journal.pgen.1004649 25275371 PMC4183435

[98] Lehnen D, Blumer C, Polen T, Wackwitz B, Wendisch VF, Unden G. 2002. LrhA as a new transcriptional key regulator of flagella, motility and chemotaxis genes in Escherichia coli. Mol Microbiol 45:521–532. doi:10.1046/j.1365-2958.2002.03032.x 12123461

[99] Shin S, Park C. 1995. Modulation of flagellar expression in Escherichia coli by acetyl phosphate and the osmoregulator OmpR. J Bacteriol 177:4696–4702. doi:10.1128/jb.177.16.4696-4702.1995 7642497 PMC177235

[100] Stojiljkovic I, Bäumler AJ, Hantke K. 1994. Fur regulon in gram-negative bacteria. Identification and characterization of new iron-regulated Escherichia coli genes by a fur titration assay. J Mol Biol 236:531–545. doi:10.1006/jmbi.1994.1163 8107138

[101] Shin J-E, Lin C, Lim HN. 2016. Horizontal transfer of DNA methylation patterns into bacterial chromosomes. Nucleic Acids Res 44:4460–4471. doi:10.1093/nar/gkw230 27084942 PMC4872104

[102] Finnerty‐Haggerty L, Knapp R, Kambhampati O, Stensland S, Kaur J, Militello KT. 2018. The role of the Escherichia coli dcm gene in stationary phase fitness and catalase activity. FASEB J 32:787. doi:10.1096/fasebj.2018.32.1_supplement.787.16

[103] Campbell JL, Kleckner N. 1990. E. coli oriC and the dnaA gene promoter are sequestered from dam methyltransferase following the passage of the chromosomal replication fork. Cell 62:967–979. doi:10.1016/0092-8674(90)90271-f 1697508

[104] Ogden GB, Pratt MJ, Schaechter M. 1988. The replicative origin of the E. coli chromosome binds to cell membranes only when hemimethylated. Cell 54:127–135. doi:10.1016/0092-8674(88)90186-9 2838178

[105] Fang G, Munera D, Friedman DI, Mandlik A, Chao MC, Banerjee O, Feng Z, Losic B, Mahajan MC, Jabado OJ, Deikus G, Clark TA, Luong K, Murray IA, Davis BM, Keren-Paz A, Chess A, Roberts RJ, Korlach J, Turner SW, Kumar V, Waldor MK, Schadt EE. 2012. Genome-wide mapping of methylated adenine residues in pathogenic Escherichia coli using single-molecule real-time sequencing. Nat Biotechnol 30:1232–1239. doi:10.1038/nbt.2432 23138224 PMC3879109

[106] Pollak AJ, Reich NO. 2012. Proximal recognition sites facilitate intrasite hopping by DNA adenine methyltransferase. J Biol Chem 287:22873–22881. doi:10.1074/jbc.M111.332502 22570478 PMC3391122

[107] Barras F, Marinus MG. 1988. Arrangement of Dam methylation sites (GATC) in the Escherichia coli chromosome. Nucleic Acids Res 16:9821–9838. doi:10.1093/nar/16.20.9821 3054812 PMC338781

[108] Smith DW, Garland AM, Herman G, Enns RE, Baker TA, Zyskind JW. 1985. Importance of state of methylation of oriC GATC sites in initiation of DNA replication in Escherichia coli. EMBO J 4:1319–1326. doi:10.1002/j.1460-2075.1985.tb03779.x 3891329 PMC554343

[109] Gottesman S, Storz G. 2011. Bacterial small RNA regulators: versatile roles and rapidly evolving variations. Cold Spring Harb Perspect Biol 3:a003798. doi:10.1101/cshperspect.a003798 20980440 PMC3225950

[110] Wang X, Wood TK. 2011. IS5 inserts upstream of the master motility operon flhDC in a quasi-Lamarckian way. ISME J 5:1517–1525. doi:10.1038/ismej.2011.27 21390082 PMC3160685

[111] Sanchez-Torres V, Hu H, Wood TK. 2011. GGDEF proteins YeaI, YedQ, and YfiN reduce early biofilm formation and swimming motility in Escherichia coli. Appl Microbiol Biotechnol 90:651–658. doi:10.1007/s00253-010-3074-5 21181144 PMC3158426

[112] Boehm A, Kaiser M, Li H, Spangler C, Kasper CA, Ackermann M, Kaever V, Sourjik V, Roth V, Jenal U. 2010. Second messenger-mediated adjustment of bacterial swimming velocity. Cell 141:107–116. doi:10.1016/j.cell.2010.01.018 20303158

[113] Park M, Patel N, Keung AJ, Khalil AS. 2019. Engineering epigenetic regulation using synthetic read-write modules. Cell 176:227–238. doi:10.1016/j.cell.2018.11.002 30528434 PMC6329643

[114] Xu X, Tao Y, Gao X, Zhang L, Li X, Zou W, Ruan K, Wang F, Xu G-L, Hu R. 2016. A CRISPR-based approach for targeted DNA demethylation. Cell Discov 2:16009. doi:10.1038/celldisc.2016.9 27462456 PMC4853773

