# Supplementary material for: DNA methylation affects gene expression but not global chromatin structure in Escherichia coli
Source: J Bacteriol. 2025 Jul 14;207(8):e00540-24. doi: 10.1128/jb.00540-24 (PMC12369378; doi:10.1128/jb.00540-24)
Supplement: Supplemental figures legends — Legends for Fig. S1 to S8. [file jb.00540-24-s0001.pdf]

SUPPLEMENTARY FIGURE CAPTIONS

Supplementary Figure 1: Gene set enrichment analysis for RNA-seq data across the indicated genotypes (relative to wild type). RNAseq data was analyzed using Rockhopper to produce q-values which assess statistical significance in expression change of each gene between strains. Directionality for expression change, where positive values indicate higher expression in the mutant relative to wild-type, was applied to the magnitudes of the log10(q-values). The values are divided into 21 evenly populated bins. iPAGE reports the representation of directional log10(q-values) across the genes annotated with each Gene Ontology (GO) term – thus, a redder bin indicates an over-representation of genes from the specified GO-term (row) at that expression change bracket (column).

Supplementary Figure 2: Distribution of changes in mean IPOD-HR or RNAP-ChIP occupancy scores in 104 bp windows centered on each Dcm target motif; positive scores indicate higher occupancy in the indicated mutant relative to WT. "Site Density" on the x-axis refers to the number of Dcm sites within each window. Asterisks represent p-values of < 0.01 by Wilcoxon signed rank test with Bonferroni correction. At the higher site densities (4 site density) there is a lack of statistical power due to a small number of loci with such high methylation site densities.

Supplementary Figure 3: Symmetrized Overlap Distances calculated as described in Amemiya *et al.*, 2022[47] to assess similarity in RNAP-ChIP occupancy peak locations between strains. A value of 0 indicates that all EPODs between the two strains overlap.

Supplementary Figure 4: Genomic context of **(A)** *selB*, **(B)** *prpE*, and **(C)** *recBD* showing 51 bp rolling mean of RNAseq reads per tens of millions of reads (Transcripts (PTMR)) that were aligned to the positive (green occupancy trace) and negative (purple occupancy trace) strands. Brown boxes above markers on the "Dam Sites" tracks indicate "7 Dam Site Density" clusters of interest. Genes are differentially colored based on their membership to functional gene clusters.

Supplementary Figure 5: Normalized read ends, as calculated by the count of read ends at each genomic position divided by the total number of million read ends within each sample, around the "7 Dam Site Density" at the **(A)** *selB*, **(B)** *prpE*, **(C)** *recB*, and **(D)** *recD* loci in each of 3 replicates for each genotype.

Supplementary Figure 6: (A) Genomic context of *flgN* showing 512 bp rolling mean of IPOD-HR (blue occupancy trace) or RNAP-ChIP (red occupancy trace) robust z-scores. Brown boxes above markers on the "Dam Sites" tracks indicate "6 Dam Site Density" clusters of interest. Genes are differentially colored based on their membership to functional gene clusters. The dashed box designates the locus which is shown in panel B. **(B)** Genomic locus of *flgN* showing IPOD-HR (blue occupancy trace) or RNAP-ChIP (red occupancy trace) robust z-scores.

Supplementary Figure 7: Heatmap representing the expression change in the regulon of regulators of *flhC*. We take the log ratio of mutant and wild-type expression values generated by Rockhopper for each gene in the regulon of the indicated *flhC* regulator. Directionality is then applied to determine whether expression changes in the regulon are consistent with the regulatory mode and expression of the regulator. E.g., genes that decrease in expression and are repressed by their regulator are "concerted" and thus contribute positively to the averaged log-fold change in expression.

Supplementary Figure 8: Semilog plots of the log_2fold-change in OD600 measurements relative to the first OD600 measurement in log phase, for the strains used in the IPOD-HR and RNAseq experiments. Lines were fit with a linear model accounting for offset between replicates.
